# A Helminth-Derived Chitinase Structurally Similar to Mammalian Chitinase Displays Immunomodulatory Properties in Inflammatory Lung Disease

**DOI:** 10.1155/2021/6234836

**Published:** 2021-11-25

**Authors:** Friederike Ebner, Katja Lindner, Katharina Janek, Agathe Niewienda, Piotr H. Malecki, Manfred S. Weiss, Tara E. Sutherland, Arnd Heuser, Anja A. Kühl, Jürgen Zentek, Andreas Hofmann, Susanne Hartmann

**Affiliations:** ^1^Institute of Immunology, Department of Veterinary Medicine, Freie Universität Berlin, Berlin, Germany; ^2^Charité-Universitätsmedizin Berlin, Freie Universität Berlin, Humboldt-Universität zu Berlin, and Berlin Institute of Health, Institute of Biochemistry, Shared Facility for Mass Spectrometry, Berlin, Germany; ^3^Macromolecular Crystallography (HZB-MX), Helmholtz-Zentrum Berlin, Berlin, Germany; ^4^Department of Structural Biology of Prokaryotic Organisms, Institute of Bioorganic Chemistry, Polish Academy of Science, Poland; ^5^Lydia Becker Institute of Immunology and Inflammation, Faculty of Biology, Medicine and Health, Manchester Academic Health Science Centre, University of Manchester, Manchester, UK; ^6^Max Delbrück Center for Molecular Medicine (MDC), Pathophysiology Platform, Berlin, Germany; ^7^Charité-Universitätsmedizin Berlin, Freie Universität Berlin and Humboldt-Universität zu Berlin, iPATH.Berlin, Berlin, Germany; ^8^Institute of Animal Nutrition, Department of Veterinary Medicine, Freie Universität Berlin, Berlin, Germany; ^9^Department of Veterinary Biosciences, Melbourne Veterinary School, The University of Melbourne, Parkville, Victoria 3010, Australia

## Abstract

Immunomodulation of airway hyperreactivity by excretory-secretory (ES) products of the first larval stage (L1) of the gastrointestinal nematode *Trichuris suis* is reported by us and others. Here, we aimed to identify the proteins accounting for the modulatory effects of the *T. suis* L1 ES proteins and studied six selected *T. suis* L1 proteins for their immunomodulatory efficacy in a murine OVA-induced allergic airway disease model. In particular, an enzymatically active *T. suis* chitinase mediated amelioration of clinical signs of airway hyperreactivity, primarily associated with suppression of eosinophil recruitment into the lung, the associated chemokines, and increased numbers of RELM*α*^+^ interstitial lung macrophages. While there is no indication of *T. suis* chitinase directly interfering with dendritic cell activation or antigen presentation to CD4 T cells, treatment of allergic mice with the worm chitinase influenced the hosts' own chitinase activity in the inflamed lung. The three-dimensional structure of the *T. suis* chitinase as determined by high-resolution X-ray crystallography revealed high similarities to mouse acidic mammalian chitinase (AMCase) but a unique ability of *T. suis* chitinase to form dimers. Our data indicate that the structural similarities between the parasite and host chitinase contribute to the disease-ameliorating effect of the helminth-derived chitinase on allergic lung inflammation.

## 1. Introduction

Chronic helminth infections are known to be accompanied by various immunomodulatory processes. The active modulation of host immune responses is believed to ensure long-term persistence of the parasites and most likely involves proteins released by the worms (reviewed in [1–3]). Over the last decade, research has focused on identifying secreted helminth immunomodulators and mechanisms of suppression in order to understand natural infection and in parallel with the aim to exploit this knowledge for the benefit of unwanted human inflammatory diseases.

We and others have identified secreted products with immunosuppressive effects in autoimmune and inflammatory diseases from diverse parasitic helminths such as *Acanthocheilonema viteae* [4–11], *Schistosoma* [12–15], *Fasciola hepatica* [16, 17], *Heligmosomoides polygyrus* [18, 19], and *Wuchereria bancrofti* [20, 21]. Among the organisms currently explored for helminth-induced therapy of humans are the pig whipworm (*Trichuris suis*, TSO), the human hookworm (*Necator americanus*, NC), and also the human whipworm (*T. trichiura*, TTO). As *T. suis* can be easily maintained in pigs but cannot multiply in humans, its administration as live parasite (TSO therapy) has been tested in a variety of clinical studies to improve human immune disorders (reviewed in [22]). Early success stories of treatments of ulcerative colitis (UC) and Crohn's disease (CD) with TSO [23–25] were followed by larger trials with only modest results for various autoimmune diseases [22, 26–28] and implicated the need for a greater understanding for the immunomodulatory mechanisms. In a rather small cohort of MS patients receiving controlled TSO treatment, we demonstrated varying degrees of parasite-specific T cell responses and cellular functionality across individuals receiving treatment, indicating the need to understand responders and nonresponders in order to develop more tailored treatment regimens [29].

*T. suis* has a direct life cycle. Upon ingestion of larval stage 1 (L1) containing infective eggs, the larvae hatch and invade the mucosa of the large intestine and develop into adults by molting several times [30]. When administered therapeutically to humans, there is evidence for a self-limiting, sterile colonization of *T. suis* [23]; thus, repeated TSO treatments are required. However, this transient exposure highlights the role of early larval stages to possess immunomodulatory properties. Using soluble products (*Ts*-SPs) or excretory-secretory proteins (*Ts*-ES) or isolated fractions from both adult worms and larval stages, several immunomodulatory mechanisms have been investigated [31–34]. *Ts*-SPs from adult worms have been shown to interfere with activation of human dendritic cells and macrophages [32, 35–38]. We have previously reported on the immunomodulatory activity of proteins collected from Ts L1 which interfere with CD4^+^ T cell priming and clinical parameters of experimental airway hyperreactivity [34]. Similarly, a recent study shows that *Ts*-ES proteins released by adult and larval stages of *T. suis* possess anti-inflammatory properties. Indeed, the authors identified a subset of proteins with anti-inflammatory properties within adult *Ts*-ES [31]. However, immunoregulators of the very early larval stages of *T. suis* remain elusive.

Here, we focus on the identification and characterization of proteins selectively secreted by the first larval stages of *T. suis*. We screened a set of *n* = 6 recombinant larval proteins for immunomodulatory properties and identified an enzymatically active *T. suis* chitinase that interfered with recruitment of immune cells and inflammatory reactions in a murine model of allergic airway disease. While our *in vitro* data suggest no direct immunomodulatory effects on, e.g., DC function or T cell activation, we observed disease amelioration to be associated with suppression of eosinophil recruitment into the lung and increased numbers of alternatively activated RELM*α*^+^ lung macrophages. X-ray crystallography revealed the structure of the nematode enzyme to be highly similar to the host's own chitinases. Since mammalian host chitinases, both true chitinases with enzymatic activity (AMCase and chitotriosidase) and chitinase-like protein (CLP) molecules which can bind to chitin but lack enzymatic activity, are implicated as either mediators or biomarkers involved in airway inflammation and fibrosis, disease amelioration by treating with a worm chitinase was rather surprising. Our findings further reveal that *T. suis* chitinase treatment of allergic mice not only inhibited host AMCase expression on mRNA level but also interfered with host chitinase activity in the BAL fluid, suggesting an alternative mechanism that might involve the high degree of structural similarity between host and nematode chitinases.

## 2. Materials and Methods

### 2.1. Ethics Statement

Mice were cared for in accordance with the principles outlined in the European Convention for the Protection of Vertebrate Animals used for Experimental and other Scientific Purposes and the German Animal Welfare Law. The mouse study was approved by the State Office of Health and Social Affairs Berlin, Germany (Landesamt für Gesundheit und Soziales; approval numbers G0144/10, T0407/17, and G0068/16).

The *T. suis* life cycle was maintained in pigs (*Sus scrofa*) in accordance with the principles outlined in the European Convention for the Protection of Vertebrate Animals used for Experimental and other Scientific Purposes and the German Animal Welfare Law. The generation of larval material was approved by the State Office of Health and Social Affairs Berlin, Germany (Landesamt für Gesundheit und Soziales; approval number H0296/12).

### 2.2. Animals

Female BALB/c mice were purchased from Janvier Labs (Le Genest-Saint-Isle, France) and maintained together with DO11.10 (OVA-TCR tg) mice at the Institute of Immunology, Department of Veterinary Medicine in Berlin. DO11.10 were typed for expression of the transgenic (tg) T cell receptor (TCR) using flow cytometry, and only mice with more than 90% of the CD4^+^ T cells expressing the OVA-tg TCR were used in the study. The *T. suis* life cycle was maintained in pigs (*Sus scrofa*) bred and cared for at the Institute of Animal Nutrition, Department of Veterinary Medicine in Berlin.

### 2.3. Murine OVA-Induced Allergic Airway Disease

Allergic airway inflammation was induced as previously described [4]. Briefly, female BALB/c mice (8 weeks old) were sensitized on days 0 and 14 with 20 *μ*g ovalbumin protein (OVA, grade VI, Sigma-Aldrich Chemie GmbH, Munich, Germany) emulsified in 2 mg alum (Imject™ Alum, ThermoFisher Scientific, IL, USA). Nonallergic control animals (PBS) received equal volumes of PBS on days 0 and 14 instead of OVA. Mice were treated with 20 *μ*g recombinant, intact *T. suis* proteins or heat-inactivated proteins on days 0, 7, and 14 i.p. or left untreated. Intranasal OVA challenge (50 *μ*g OVA/25 *μ*l PBS) was performed on all groups of mice on days 28 and 29. Where indicated, lung function was assessed on day 30 at the Max Delbrück Center for Molecular Medicine (MDC) in Berlin. On day 31, mice were sacrificed, bronchoalveolar lavage (BAL) was performed, and samples were taken for analysis.

### 2.4. Airway Hyperreactivity

Airway resistance and lung function were assessed via double-chamber plethysmography (emka Technologies, Paris, France) in response to increasing doses of acetyl-ß-methylcholine (Methacholine, Sigma-Aldrich Chemie GmbH, Munich, Germany).

Mice were placed into a restrainer without anesthesia and adjusted (5 min), and the following protocol was applied: baseline measurement (4 min), PBS (1 min), measurement (4 min), 6.25 mg/ml methacholine (1 min), measurement (4 min), 12.5 mg/ml methacholine (1 min), measurement (4 min), 25 mg/ml (1 min) methacholine, measurement (4 min), 50 mg/ml methacholine (1 min), and measurement (4 min).

Data were sampled once per second. For data analysis, averages of 5 sec intervals were used. Airway resistance (*S*_raw_) and expiratory time values were generated and analyzed using iox2 software (iox v2.8.0.13, emka Technologies). Resulting data points were averaged again per mouse and methacholine dose. Data points with success rate < 50% and beat number < 10 were excluded from the analysis.

### 2.5. Bronchoalveolar Lavage

Intratracheal bronchoalveolar lavage (BAL) was performed on i.p. anaesthetized mice by inflating the lungs with 800 *μ*l PBS containing protease inhibitor cocktail (cOmplete™, Mini, EDTA-free Protease Inhibitor, Roche Diagnostics GmbH, Mannheim, Germany). The 1^st^ BAL was pelleted, and the supernatant was stored at -20°C for cytokine/chemokine detection. Four additional lavages (4 × 800 *μ*l PBS) were performed for cell isolation and pelleted by centrifugation. All cells were pooled in 500 *μ*l PBS and counted using a Neubauer cell chamber. 2 × 100 *μ*l cell suspension per sample was used for cytospin preparation. Object slides were dried and stained with Diff-Quick solutions (Labor und Technik Eberhard Lehmann GmbH, Berlin, Germany) for differential cell counts. Residual cells were used for flow cytometry.

### 2.6. Histology and Immunohistochemistry

Following lung lavage, the left lobe was split into two pieces. The lower tissue was degassed and fixed in a formalin solution (Roti-Histofix 10%, Carl Roth GmbH + Co. KG, Karlsruhe, Germany) for 6 h at room temperature and then stored at 4°C. The tissue was embedded in paraffin, and sections were stained with an antibody against RELM-*α* (polyclonal rabbit anti-mouse, Abcam, Cambridge, UK). Nuclei were stained with hematoxylin (Merck). Interstitial RELM-*α*^+^ cells were quantified in 5 HPF (high-power fields, 400x magnification). Pictures are presented at 400x magnification. For evaluation of histomorphological changes, paraffin sections were stained with hematoxylin and eosin (H&E) and scored for inflammation and goblet cell hyperplasia. Briefly, score 1 is for minor perivascular inflammation, score 2 for moderate perivascular and peribronchial inflammation and minimal goblet cell hyperplasia, score 3 for increased perivascular and peribronchial inflammation and increased goblet cell hyperplasia (beginning in smaller airways), and score 4 for severe perivascular, peribronchial, and interstitial inflammation with goblet cell hyperplasia in smaller and larger airways.

### 2.7. Generation of Bone Marrow-Derived Dendritic Cell (DC) and Activation Assay

Isolated bone marrow cells from the tibia and femur of female BALB/c mice were seeded at 5 × 10^5^ cells/ml in 10 ml RPMI-1640 media (PAN-Biotech) containing 10% fetal calf serum (FCS), 20 mM *L*-glutamine, 100 U/ml penicillin, and 100 *μ*g/ml streptomycin (PAN-Biotech) and differentiated in the presence of recombinant GM-CSF (20 ng/*μ*l) (PeproTech). At days 6–8, nonadherent cells were collected. For studying effects of *Ts*-Chit on DC activation, BM-DCs were preincubated with 10 *μ*g/ml recombinant *Ts-*Chit protein for 2.5 h and stimulated with 100 ng/ml LPS-B5 (Sigma-Aldrich Chemie GmbH, Munich, Germany) or 1 *μ*M CpG (ODN 1668, InvivoGen, CA, USA) for additional 20 h. Cell culture supernatants were collected and stored at -20°C for cytokine ELISA, and DCs were analyzed for activation markers CD86, MHCII, and Ox40-L by flow cytometry.

### 2.8. T Cell Proliferation Assays *In Vitro*

For studying effects of *Ts-*Chit on antigen-independent T cell proliferation, splenocytes were isolated from female BALB/c mice, labeled with CFSE in PBS (8 min, RT, 5 *μ*M), and seeded at a density of 4 × 10^6^ cells/ml in round-bottom 96-well plates. After 4 days of incubation (37°C, 5% CO_2_) in the presence of plate-bound mouse *α*CD3/*α*CD28 (1 *μ*g/ml) and recombinant *Ts-*Chit (10 *μ*g/ml), DCs were harvested and analyzed for CFSE dilution in CD4^+^ and CD8^+^ T cells.

For studying effects of *Ts-*Chit on antigen-dependent T cell proliferation and differentiation, MACS-purified OVA-TCR tg CD4^+^ T cells isolated from DO11.10 mice were labeled with CFSE (5 *μ*M, eBioscience) in PBS for 8 min at room temperature. CFSE-labeled CD4^+^ T cells (4 × 10^5^) were cocultured with OVA-loaded and *Ts*-Chit pretreated BM-DC (1 × 10^5^) for 4 days and analyzed by flow cytometry for CFSE dilution and Treg/Th transcription factors.

### 2.9. Multiplex Assay

Bronchoalveolar lavage supernatants were analyzed for cytokine and chemokine levels using the Mouse Cytokine & Chemokine 26-Plex ProcartaPlex™ Panel (ThermoFisher, MA, USA) according to the manufacturer's instructions. The MAGPIX™ instrument (Luminex) was used, and data were analyzed with ProcartaPlex™ Analyst 1.0.

### 2.10. Flow Cytometry

The following monoclonal fluorochrome conjugated anti-mouse antibodies were used: CD11b (clone M1/70, APC-Cy7), CD64 (clone X54-5/7.1, PE-Cy7), IgG1 (clone RMG1-1, APC), and IgG2a (clone RMG2a-62, PerCP-Cy5.5) were purchased from BioLegend, CA, USA. CD45 (clone 30-F11, eF450), MHC-II (clone M5/114.15.2, PE-Cy5), Gr1 (clone RB6-8C5, PE-Cy7), and EpCAM (clone G8.8, APC) were purchased from eBioscience, CA, USA. CD11c (clone HL3, FITC), MHC-II (clone M5/114.15.2, PE), CD86 (clone GL1, Pe-Cy7), IL-13 (clone eBio13A, A488), CD4 (clone RM4-5, Horizon V500), GATA3 (clone TWAJ, A660), Foxp3 (clone FJK-16s, PE), Tbet (4B10, BV 421), and SiglecF (clone E50-2440, PE) were purchased from BD Biosciences, CA, USA. Viability dye in eFluor 506, eF660, and eF780 and streptavidin (FITC) were purchased from eBioscience, CA, USA. Polyclonal biotinylated anti-mouse RELM-*α* was purchased from PeproTech, NJ, USA. Samples were acquired using Canto II (BD Biosciences, CA, USA) and analyzed using FlowJo software v9, Tristar.

### 2.11. Quantitative RT-PCR

Following lung lavage, the upper right lobe was snap-frozen in liquid nitrogen and stored at -80°C. RNA isolation from lung tissues was performed using the innuPREP RNA Mini Kit (Analytic Jena AG, Jena, Germany) according to the manufacturer's instructions. Reverse transcription of 1 *μ*g RNA into cDNA was done using the High-Capacity cDNA Reverse Transcription Kit (Applied Biosystems, CA, USA). RT-PCR was performed on the LightCycler® 480 instrument using the LightCycler® 480 SYBR Green I Master Mix (Roche Diagnostics, Mannheim, Germany) and oligonucleotides listed in Table 1. Gene expression is described relative to the housekeeping gene peptidylprolyl isomerase A (PPIA) and normalized to mRNA levels of the PBS group if not indicated differently.

### 2.12. Isolation of *T. suis* Larvae and Production of Excretory-Secretory (ES) Proteins

Pigs were orally infected with 8000 embryonated *T. suis* eggs in 2 ml NaCl (0.9%). Following euthanasia on days 10, 18, and 28 dpi, *T. suis* larvae were manually collected from the caecum and colon. Larvae were washed 4 times for 15 min each in RPMI-1640 (PAN-Biotech, Aidenbach, Germany) supplemented with 200 U/ml penicillin, 200 *μ*g/ml streptomycin, and 1.35 *μ*g/ml amphotericin B (all purchased from PAN-Biotech) by sedimentation and incubated overnight in RPMI-1640 supplemented with 1% glucose (Sigma-Aldrich, St. Louis, US), 100 U/ml penicillin, 100 *μ*g/ml streptomycin, and 0.625 *μ*g/ml amphotericin B. Culture medium was replaced, and larvae were incubated for another 5 days while conditioned media were collected every 2^nd^ day, sterilized by filtration (0.45 *μ*m Minisart Syringe Filter, Sartorius AG, Göttingen, Germany), and stored at -20°C.

*T. suis* L1 ES proteins were isolated from freshly hatched larvae as previously described [34]. Identification of *T. suis* larva ES proteins (*in vitro*-hatched L1, 10 dpi, 18 dpi, and 28 dpi) was done by mass spectrometry as previously described [34]. In brief, TCA-precipitated dried protein pellets of *T. suis* were suspended, reduced, alkylated, and tryptic digested. The resulting peptides were analyzed by LC-MSMS on a 4700 Proteomics Analyzer (ABSCIEX, Framingham, MS). Database searches were performed with Mascot software against NCBI database 20150331 (64057457 sequences). Proteins were accepted as identified if at least two MS/MS spectra provided a MASCOT score for identity (*p* < 0.01).

### 2.13. Recombinant Protein Expression

*T. suis* mRNA was isolated out of 10-20 mg worms by using the innuPREP RNA Mini Kit (Analytic Jena AG, Jena, Germany) according to manufacturer's instructions. Reverse transcription of 1 *μ*g RNA into cDNA was done using the Transcriptor First Strand cDNA Synthesis Kit (Roche Diagnostics GmbH, Mannheim, Germany).

The genes corresponding to selected *T. suis* proteins were amplified and cloned into the vector pLEXSYsat2 of the Leishmania expression system (LEXSYcon2 Expression Kit, Jena Bioscience GmbH, Jena, Germany). The cloning vector was sequenced for verification. *Leishmania tarentolae* cells were transfected via electroporation (Nucleofector™ 2b Device, Amaxa™ Human T Cell Nucleofector™ Kit, Lonza, Basel, Switzerland) with the respective expression cassette containing the selective antibiotic marker nourseothricin (NTC) and the *T. suis* protein-coding gene. Transfected *L. tarentolae* cells were polyclonally selected and maintained as described in the manual.

### 2.14. Protein Purification

His_6_-tag fused, recombinant proteins secreted by transfected *Leishmania* cells were purified by liquid chromatography over His-Trap™ excel columns (GE Healthcare, Buckinghamshire, United Kingdom). Protein-containing fractions were pooled and dialyzed two times against PBS (4 h and overnight). Finally, protein concentrations were assessed using Pierce™ BCA Protein Assay Kit (ThermoFisher Scientific, Rockford, USA). Identity of purified, recombinant proteins was verified by LC-MSMS analysis after tryptic digestion.

### 2.15. SDS-PAGE

To validate recombinant expression of *T. suis* proteins, a 12% SDS gel was loaded with 1-2 *μ*g purified, recombinant *T. suis* proteins and developed using silver staining (Pierce Silver Stain Kit, ThermoFisher Scientific, Rockford, US).

### 2.16. Chitinase Assay

Chitinase activity was analyzed by using the substrate and standard solutions of the fluorimetric chitinase assay kit (Sigma-Aldrich Chemie GmbH, Munich, Germany). Substrates 4-MU-(GlcNAc)_2_ (exochitinase activity) and 4-MU-(GlcNAc)_3_ (endochitinase activity) were adjusted to 1.1 mM in DMSO and diluted 1 : 20 in assay buffer (100 mM citric acid, 200 mM sodium phosphate, pH 5.6) immediately prior to starting the assay by adding 50 *μ*l of diluted substrate solution to 10 *μ*l of protein KFD48490.1 (*Ts-*Chit) (5 *μ*g/ml). The reaction mix was incubated for 30 min at 37°C and then quenched by adding 500 *μ*l stop solution (500 mM sodium carbonate, 500 mM sodium bicarbonate, pH 10.7). 250 *μ*l of the reaction solution was transferred in duplicate into a black 96-well plate, and fluorescence of liberated 4-MU was measured using an excitation wavelength of 360 nm and recording emission at 450 nm. The effect of pH on KFD48490.1/*Ts-*Chit chitinase activity was determined by using assay buffers adjusted to pH = 2, 3, 4, 5, 6, 7, 8, 9, and 10. Heat stability of *Ts-*Chit chitinase was assessed by preincubation of the protein for 60 min at different temperatures (4°C–90°C) followed by the chitinase assay.

### 2.17. Crystallization and X-Ray Data Collection

The recombinant protein solution was dialyzed after initial purification in 20 mM HEPES buffer (pH 7.5) and purified over a HiLoad 16/600 Superdex 200 pg column with a HEPES running buffer (20 mM HEPES pH 7.7, 200 mM NaCl, and 1 mM TCEP). Elution fractions were concentrated using Amicon Ultra-4 concentrator tubes (30,000 MWKO) to a final concentration of 33 mg/ml. A Gryphon pipetting robot was used to set up crystallization plates using the JSCG-plus Screen by mixing 0.25 *μ*l protein solution with 0.25 *μ*l crystallization cocktail solution. Plates were sealed and incubated at 20°C. Crystal growth was monitored regularly under an optical microscope. The crystals appeared in condition H7 (0.8 M ammonium sulfate, 0.1 M BIS-Tris pH 5.5, and 25% (*w*/*v*) PEG 3350). Protein crystals were harvested, cryoprotected using reservoir solution supplemented with 25% (*v*/*v*) ethylene glycol, and stored in liquid nitrogen. Diffraction data were collected on BL14.1 at the BESSY II synchrotron of the Helmholtz-Zentrum Berlin [39] and processed using XDSAPP [40]. Relevant processing statistics are shown in Table 2. The structure was solved by molecular replacement using the structure of human chitotriosidase (CHIT1) as a search model (PDB-Id 5HBF, [41]). After several cycles of manual rebuilding using COOT [42] and refinement using REFMAC5 [43], refinement converged at *R* factors of 19 and 23% for the working *R* and the free *R*, respectively (Table 3). The refined structure features good refinement statistics and excellent geometric parameters. The refined coordinates and the associated structure factor amplitudes were deposited in the PDB using the accession code 6G9C.

### 2.18. Structure-Based Sequence Alignment and Surface Electrostatics

To obtain an overview of structural similarities and differences between *Ts*-Chit and host chitinases, the topology and secondary structure elements of *Ts*-Chit were compared to six mouse chitinases and chitinase-like proteins by means of a structure-based amino acid sequence alignment (see Supplementary Information S2). Secondary structure elements were predicted with PSIPRED [44], and a structure-based amino acid sequence alignment was constructed using SBAL [45].

In order to assess whether *Ts*-Chit possesses any features that might serve as a potential molecular mimicry of host chitinases when acting as an antigen, we undertook a comparison of surface features of *Ts*-Chit with mouse chitinases. In general, this approach is limited by the availability of only one experimental 3D structure of mouse chitinases. Using the amino acid sequences of three mouse chitinases (*M. musculus* AMCase, Chit1 isoform 1, and Ym1), known 3D structures or close structural homologues of these proteins were identified in the PDB using pGenThreader [46], revealing mouse AMCase as the only mouse protein suitable for comparison in this context.

Using the monomer and dimer structures of *Ts*-Chit as well as the structure of mammalian AMCase (PDB 3FY1), surface electrostatics for these three proteins were calculated with APBS [47] and visualized with UCSF Chimera [48].

### 2.19. Statistical Analysis

Statistical analysis was done using GraphPad Prism software v7 (GraphPad Software, Ca, USA) using the Kruskal-Wallis test (Dunn's multiple comparisons test), Mann-Whitney test, or unpaired Student *t*-test as indicated. Values with *p* < 0.05 (^∗^), *p* <0.005 (^∗∗^), and *p* <0.001 (^∗∗∗^) were considered to be significant.

## 3. Results

### 3.1. *T. suis* L1 Stage-Specific Excretory-Secretory Protein KFD48490.1 Affects Allergic Airway Inflammation

**Figure 1:**
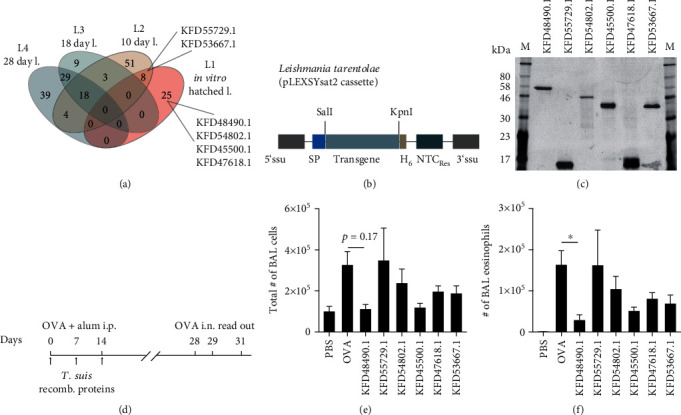
*T. suis* L1 ES proteins are highly stage-specific, and candidates reduce allergic airway hyperreactivity when recombinantly expressed and applied. (a) Protein ID counts resulting from proteomic MS-based analysis (see Supplementary Table S1) were compared across the proteomic datasets of *T. suis* L1, L2, L3, and L4 ES proteins. Shown are protein IDs selected for recombinant expression. (b) Expression cassette of LEXSY eukaryotic expression system illustrating positioning of the export signal peptide that mediates secretion (SP), the selection antibiotic (nourseothricin, NTC), and the His_6_-tag for purification (H_6_). The cassette was integrated into the *L. tarentolae* genome. (c) Silver-stained SDS/PAGE gel of recombinantly expressed *T. suis* L1 proteins. (d) Experimental setup of acute ovalbumin- (OVA-) induced allergic airway hyperreactivity model and treatment regimen with recombinant *T. suis* proteins. Animals were OVA-sensitized on days 0 and 14 and challenged with OVA intranasally on days 28 and 29. BAL fluid was collected from pretreated or untreated animals at day 31. (e) Total number of cellular infiltrates in BAL fluid. Shown are nonallergic mice (PBS controls), untreated allergic mice (OVA), and treatments with recombinant *T. suis* proteins indicated by GenBank IDs. (f) Numbers of eosinophils in BAL fluid detected by differential cell counts of cytospin preparations stained with Diff-Quick over the different treatment groups. Data shown are mean + SEM of *n* = 5–10 animals from 2 separate experiments. Statistically significant differences were determined using a Kruskal-Wallis test (Dunn's multiple comparisons test) and are indicated, ^∗^*p* ≤ 0.05.

We previously showed that *T. suis* excretory/secretory proteins (ES proteins) collected from L1 hatched *in vitro* reduced clinical signs of murine allergic airway disease [34]. To identify proteins responsible for this effect, we now investigated the protein composition of L1 ES proteins and compared it to L2, L3, and L4 larval stages of *T. suis*-released products. For stage-specific expression patterns, parasites were isolated from pigs infected with 8000 TSO at 10, 18, and 28 days postinfection (resulting in L2, L3, and L4 larvae, respectively) and cultivated to collect conditioned media for mass spectrometry. Alternatively, eggs were hatched *in vitro* to generate L1 larvae and conditioned media, as previously described [34]. Comparative proteomic analysis of *T. suis* ES proteins (Figure 1(a) and Supplementary Table S1) revealed some overlap between first stage (*in vitro* hatched) L1 larvae and L2 larvae (10 day larvae), but no overlap of L1 ES proteins with later stages of larval development (L3 ES from 18 day larvae and L4 ES from 28 day larvae). Four L1-specific proteins and two L1/L2-specific proteins with predicted signal sequences and a lack of transmembrane domains (Table 2) were recombinantly expressed. Since glycosylation can be crucial for the immunomodulatory effects of secreted helminth proteins, we used the eukaryotic expression system LEXSY, based on the protozoan *L. tarentolae* (Figure 1(b)). Notably, selected *T. suis* L1 proteins were distinct in size (ranging from 13 to 56 kDa, Figure 1(c)), predicted glycosylation sites and homology-based annotation (Table 2).

We initially screened for immunomodulatory effects of the six recombinant *T. suis* L1 proteins using the OVA- (ovalbumin-) induced allergic airway hyperreactivity model in female BALB/c mice. Mice were treated with recombinant *T. suis* proteins during sensitization with the allergen OVA on days 0, 7, and 14 (Figure 1(d)). The numbers of total BAL infiltrates and BAL eosinophils were detected 2 days after the 2^nd^ intranasal OVA challenge at day 31 (Figure 1(d)) and analyzed in parallel to control mice lacking OVA sensitization, but receiving OVA-challenged mice (PBS) and untreated allergic mice (OVA). Analysis of total cell numbers and cellular composition of the BAL fluid found that KFD48490.1 (homologous to *T. trichiura* acidic mammalian chitinase) and KFD45500.1 (homologous to *T. trichiura* venom allergen 5) reduced the total numbers of cellular BAL fluid infiltrates, but only KFD48490.1-treated mice showed a significant reduction of eosinophils infiltrating the lungs in comparison to untreated allergic (OVA) mice (Figures 1(e) and 1(f)).

### 3.2. Immunoregulatory KFD48490.1 Is an Active Chitinase of *T. suis* L1 Larvae

**Figure 2:**
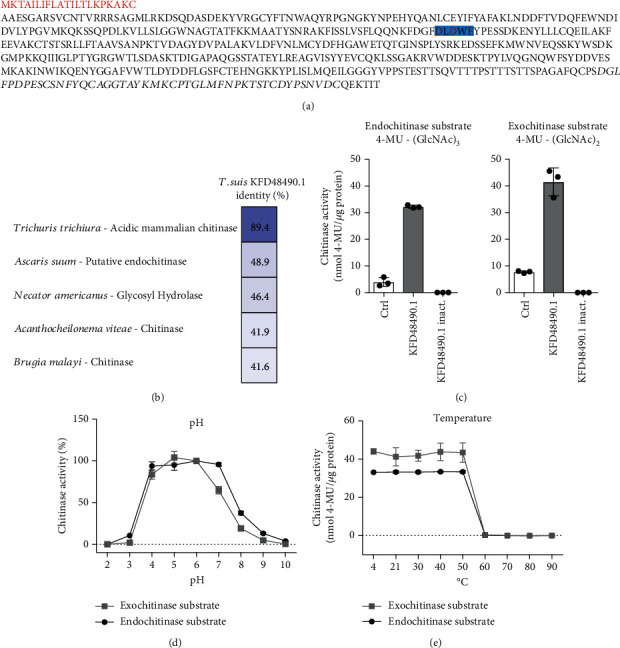
KFD48490.1 is an active chitinase. (a) KFD48490.1 amino acid sequence with mapped signal peptide (red), catalytically active residues (highlighted in blue and red), and the chitin-binding domain (black, italic). (b) Amino acid sequence alignment of KFD48490.1 and known nematode chitinases. (c) Fluorimetric chitinase assay testing recombinant KFD48490.1, heat-inactivated KFD48490.1, and a chitinase control enzyme (ctrl) with substrates for endochitinase (left) and exochitinase (right) activity. Data shown are mean ± SD of *n* = 3 replicates from one of three independent experiments. (d) Chitinase assay testing KFD48490.1 activity depending on pH (range 2-10). (e) Chitinase assay testing KFD48490.1 preincubated at different temperatures (4–90°C) for 1 h prior to the assay. Data shown are mean ± SD of *n* = 3 replicates.

Based on amino acid sequence similarity, database research predicted KFD48490.1 was homologous to acidic mammalian chitinase (AMCase) of *T. trichiura* (Table 2). The amino acid sequence of KFD48490.1 revealed the presence of the catalytic motive DxDxE (*T. suis* DLDWE, Figure 2(a)), typically observed for active chitinases, and a C-terminal chitin-binding domain with cysteine residues responsible for the chitin binding (Figure 2(a)). Alignment studies additionally proved high sequence similarities to other known helminth chitinases including a putative endochitinase from the pig infecting *Ascaris suum* and a glycosyl hydrolase from the human hookworm *N. americanus* (Figure 2(b)). Active chitinases cleave chitin from the end of a polymer chain (exochitinase) and/or hydrolyse small oligomers to generate N-acetylglucosamine monomers within the oligomer chain (endochitinase) [49]. To examine whether KFD48490.1 exerts true chitinase activity, we used two different substrates, 4-MU-(GlcNAc)_3_ and 4-MU-(GlcNAc)_2_, allowing us to evaluate both endo- and exochitinase activity, respectively (Figure 2(c)). KFD48490.1 clearly presented both, endo- and exochitinase activity, whereas extensive, heat-induced proteolysis resulted in a loss of chitinase activity (Figure 2(c)).

With regard to the biology of nematodes, we explored the influence of low pH and high temperatures hypothesizing that *T. suis* chitinase will be active at low pH and elevated temperatures in the pig's intestine to support larval hatching and development [50, 51]. Interestingly, KFD48490.1 remained enzymatically active over a wide range of pH 4–7 (Figure 2(d)) and up to temperatures of 50°C (Figure 2(e)). Together, these data confirmed the functional annotation of KFD48490.1 as *T. suis* L1 true chitinase, and thus, the protein is referred to as *Ts*-Chit from hereon.

### 3.3. *T. suis* Chitinase Does Not Act on DC Activation Marker or T Cell Proliferation

**Figure 3:**
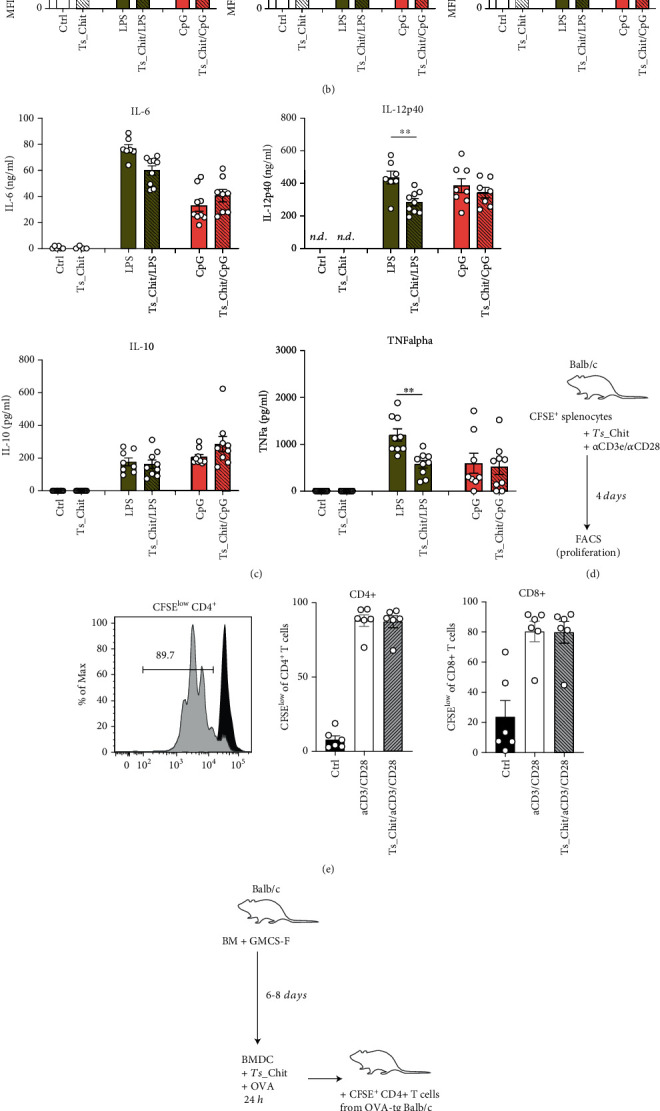
No direct effects of *T. suis* chitinase on DC activation or T cell proliferation. (a) Experimental setup of analyzing effects of *Ts-*Chit on activation marker und cytokine release from bone marrow-derived dendritic cells. (b) MFI of MHCII, CD86, and Ox40L expression analyzed by flow cytometry. Data are presented as individual values and mean ± SEM of *n* = 9 animals from *n* = 3 separate experiments. (c) Cytokine levels of IL-6, IL-12p40, IL-10, and TNF*α* of BMDC supernatants harvested 24.5 h following TLR ligation and *Ts-*Chit treatment detected by ELISA. Data are presented as individual values and mean ± SEM of *n* = 7-9 animals from *n* = 3 separate experiments. Statistical significance was assessed using a two-tailed Mann-Whitney test, and results are indicated by ^∗∗^*p* ≤ 0.01. (d) Experimental setup of analyzing effects of *Ts-*Chit on CD3e/CD28-induced T cell proliferation using CFSE-labeled splenocytes. (e) Exemplary histogram of CFSE dilution in CD4^+^ T cells with (gray) and without (black) anti-CD3e/CD28 stimulation and summarized in bar graphs for CD4 and CD8 T cell proliferation. Data are presented as individual values and mean ± SEM of *n* = 6 animals from *n* = 2 separate experiments. (f) Experimental setup of analyzing effects of *Ts-*Chit on antigen-dependent T cell proliferation and differentiation. MACS-purified CD4^+^ T cells from DO11.10 mice were cocultured with OVA-loaded BMDC in the absence or presence of *Ts-*Chit. (g) CFSE dilution of CD4^+^ T cells and transcription factor expression of Foxp3, Tbet, and GATA3 were analyzed in CFSE^low^ CD4^+^ T cell and were analyzed by flow cytometry and presented as summarized for *n* =5 mice from two independent experiments.

Direct immunomodulatory effects of ES or soluble proteins from adult and larval parasitic nematodes have previously been shown *in vitro*, and various modulatory activities were found such as targeting proinflammatory cytokine release from bone marrow-derived dendritic cells or macrophages and induction of arginase-1, IL-10, and NO in macrophages [4, 31, 36, 52, 53], the suppression of antigen-specific or unspecific CD4^+^ T cell proliferation [54], or induction of Foxp3^+^ regulatory T cells [19, 55]. Hence, we investigated *T. suis* chitinase (Ts-Chit) activity on dendritic cell activation and T cell proliferation. Recombinant *T*s-Chit did not interfere with upregulation of LPS- or CpG-induced activation markers on bone marrow-derived dendritic cells (BMDC) such as MHCII, CD86, or Ox40L (Figures 3(a) and 3(b)). While CpG-induced proinflammatory cytokine expression was not affected (Figure 3(c)), we observed reduced secretion of LPS-induced IL-12p40 and TNF*α*when BMDC were treated with *Ts*-Chit. We could also not detect induction of regulatory IL-10 in BMDC (Figure 3(c)). Similarly, TCR-unspecific proliferation of CD4^+^ and CD8^+^ T cells was not affected (Figures 3(d) and 3(e)) when cultured in the presence of *Ts-*Chit. OVA-tg CD4^+^ T cells loaded onto OVA-presenting BMDC resulted in similar proliferation of OVA-recognizing T cells in both *Ts-*Chit-treated and *Ts-*Chit-untreated cultures (Figures 3(f) and 3(g)). Again, we did not observe significant effects on T cell polarization (Figure 3(g)). Although a strong, direct immunomodulatory activity of *Ts-*Chit was not established by the *in vitro* assays that we performed, we aimed to decipher what caused the *in vivo* effects of the nematode chitinase and went back to the mouse model of AHR.

### 3.4. *T. suis* Chitinase- (*Ts*-Chit-) Mediated Regulation of Airway Hyperreactivity Only Partially Depends on Protein Integrity

To investigate the effects of *Ts*-Chit treatment on allergic airway disease, we analyzed airway resistance, expiration time, and BAL fluid profiles in more detail. In addition, we included a group of mice treated with heat-inactivated, recombinant *Ts*-Chit to assess whether immunomodulatory effects were linked to the enzymatically active *T. suis* chitinase protein.

**Figure 4:**
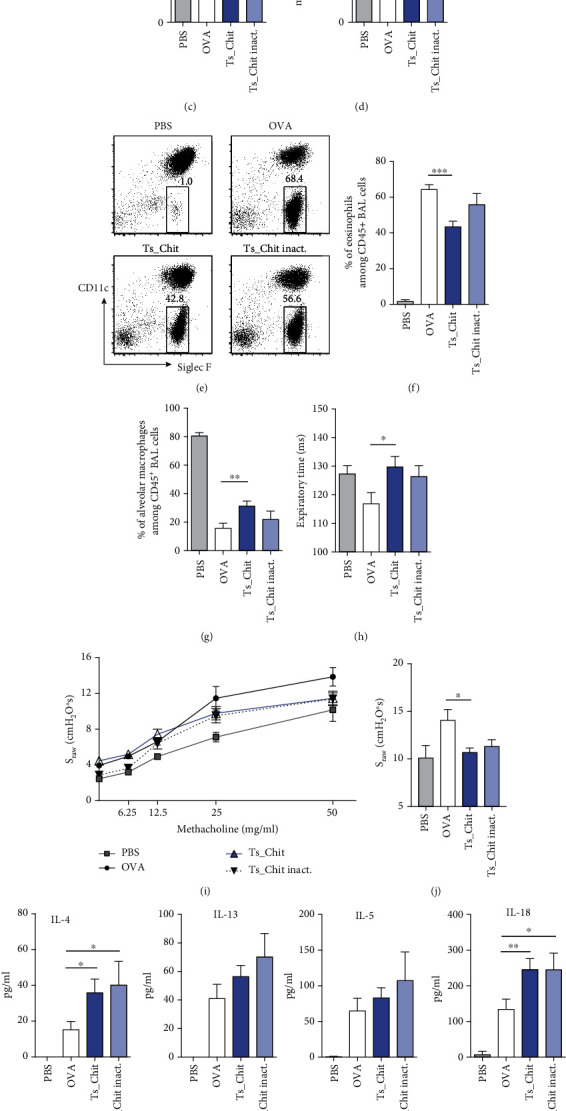
*Ts-*Chit treatment mediates attenuation of airway hyperreactivity. Bronchoalveolar lavage (BAL) was performed two days after OVA challenge, and the BAL fluid was examined for (a) total number of infiltrating cells (Neubauer chamber), (b) BAL eosinophils, (c) BAL neutrophils, and (d) monocytes/macrophages using cytospin preparations and Diff-Quik staining. (e) Representative plots of BAL-infiltrating cells using flow cytometry and focusing on eosinophil frequencies (identified as CD45^+^CD11b^+^GR1^low^CD11c^−^SiglecF^+^). Summary of flow cytometric analysis of (f) BAL eosinophils and (g) alveolar macrophages (CD45^+^CD11b^low^CD11c^+^). (h) Basal expiration time during PBS inhalation measured using whole-body double-chamber plethysmography 24 h after OVA challenge. (i) Concentration response curve of methacholine sensitivity. Specific airway resistance (*S*_raw_) was assessed in response to increasing doses of methacholine. (j) *S*_raw_ values recorded when nebulizing 50 mg/ml of methacholine. (k) BAL cytokines detected two days after challenge using bead-based multiplexing. (l) Exemplary contour plots showing IL-13/GATA3 coexpression among lung-infiltrating CD4^+^ T cells of allergic mice. Bar graph summarizes GATA3 frequency among CD4^+^Foxp3^−^ T cells of lung lymphocytes of treatment groups (*n* = 4–5 mice per group). (a–k) Data are presented as mean ± SEM of *n* = 10–12 animals from *n* = 3 separate experiments. Statistical significance was assessed using a two-tailed Mann-Whitney test, and results are indicated by ^∗^*p* ≤ 0.05, ^∗∗^*p* ≤ 0.01, and ^∗∗∗^*p* ≤ 0.001.

Treatment of animals with intact and active *Ts*-Chit significantly reduced the number of total cells (Figure 4(a)) and eosinophils (Figure 4(b)) present in BAL fluid 2 days after 2^nd^ OVA challenge. Differential cell counts of BAL fluid further revealed that neutrophil numbers were largely unaffected (Figure 4(c)). A trend towards an increase of alveolar macrophage cell numbers was observed in *Ts*-Chit compared to allergic (OVA) mice (Figure 4(d)). Flow cytometry of BAL cells confirmed the reduced frequency of eosinophils (CD45^+^CD11b^+^GR1^low^CD11c^−^SiglecF^+^) in *Ts*-Chit-treated compared to untreated (OVA) allergic animals (Figures 4(e) and 4(f)) and also supported increased frequencies of alveolar macrophages, identified as CD11b^low^CD11c^+^ cells among CD45^+^ cells (Figure 4(g)), as observed as a trend in differential cell counts. The respiratory responses resulting from *Ts*-Chit treatment were measured using noninvasive whole-body plethysmography 24 h after OVA challenge. Importantly, also, the nonsensitized control group (PBS) was intranasally challenged with OVA. Preventative administration of *Ts*-Chit caused restoration of expiration time to the level of PBS control mice at baseline (Figure 4(h)). A trend in recovery was also noticed for treating with heat-inactivated *Ts*-Chit (Figure 4(h)). Measuring airway resistance to increasing doses of methacholine (MCh) revealed that *Ts*-Chit treatment attenuated exacerbation of MCh-induced airway hyperreactivity, but treatment with inactivated *Ts*-Chit also had some effects (Figures 4(h)–4(j)). We next evaluated whether the reduced cellularity and improved lung function observed in *Ts*-Chit-treated animals were reflected in BAL cytokine levels. OVA sensitization and challenge caused a significant increase of the cytokines IL-4, IL-5, and IL-13 detected in BAL fluid; however, *Ts*-Chit treatment did not reduce but rather enhanced local Th2 cytokine production (Figure 4(k)). Of note, TNF*α* levels in BAL did not vary among groups (data not shown). Interestingly, *Ts*-Chit and heat-inactivated *Ts*-Chit alike accelerated IL-18 levels in BAL fluid (Figure 4(k)), a cytokine known to induce IL-4, IL-5, and IL-13 release by mast cells and basophils [56]. Thus, cellular infiltration and the respiratory response were improved in mice treated with *Ts*-Chit, but Th2 cytokine level in BAL was unchanged or elevated in comparison to untreated allergic mice (OVA). Phenotyping of lung inflammatory CD4 T cells revealed elevated frequencies of IL-13-producing and GATA3-expressing Th2 cells in mice receiving *Ts*-Chit or inactivated *Ts*-Chit (Figure 4(l)), supporting our findings on enhanced local Th2 responses in chitinase-treated mice independent from the functional integrity of the nematode chitinase.

**Figure 5:**
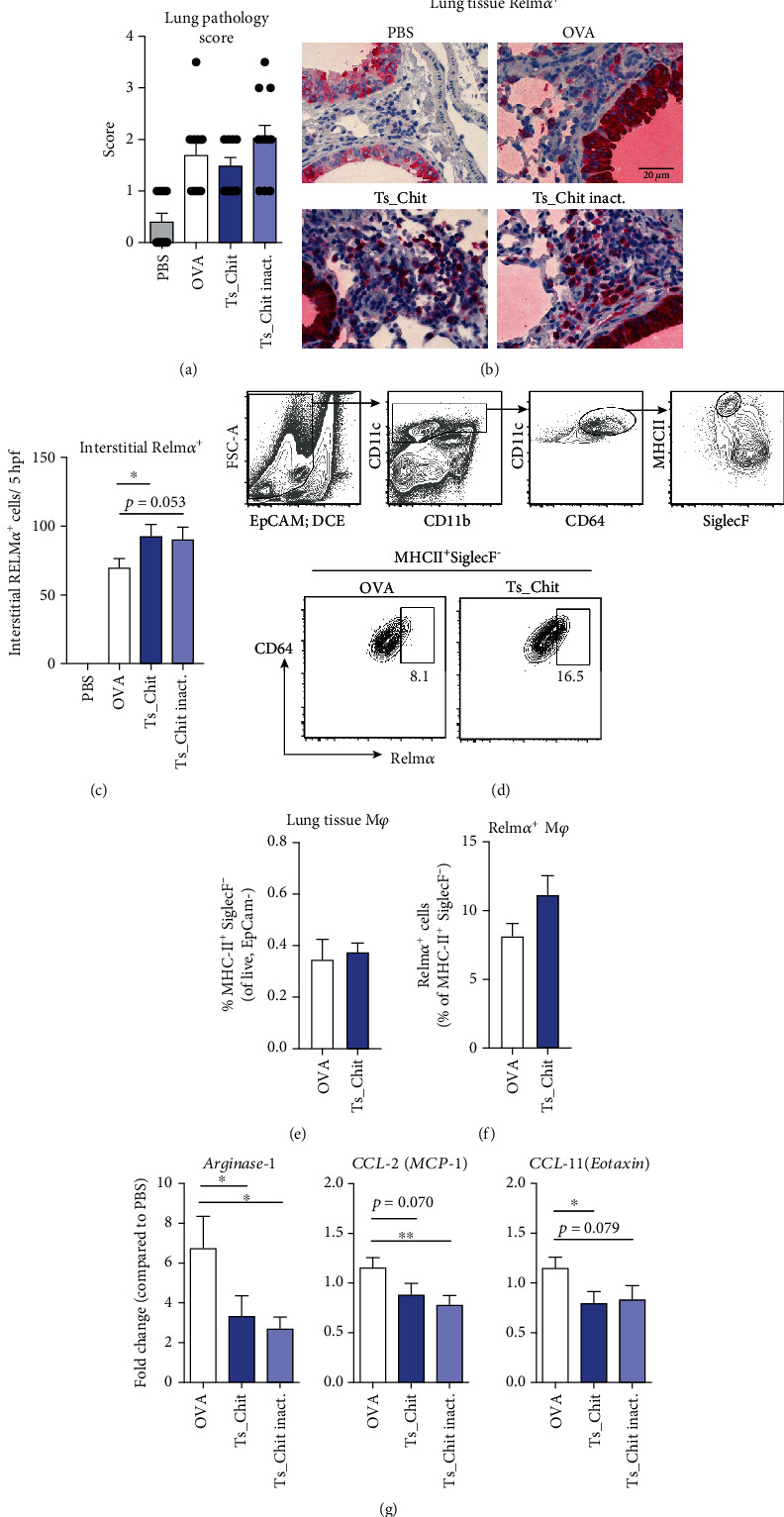
*Ts*-Chit-mediated effects on lung parenchymal macrophages. (a) Histopathological evaluation of tissue inflammation. (b) Histological sections of paraffin-embedded lung tissues from treated (*Ts*-Chit and *Ts*-Chit inact.), untreated (OVA), and nonsensitized (PBS) controls stained for RELM*α* (red) and hematoxylin (steel blue) and presented at 400× magnification. (c) Histological quantification of interstitial RELM*α*^+^ cells. (d) Gating strategy and representative plots of RELM*α*^+^ macrophages (EpCAM^−^CD11c^+^CD64^+^MHCII^+^Siglec^−^) of lung homogenates from OVA-sensitized and challenged mice with or without *Ts*-Chit treatment summarized in (e) and (f). (g) mRNA expression of *arginase-1*, *monocyte chemotactic protein 1* (*MCP1/CCL-2*), and *CCL-11/eotaxin* in lung tissue samples of mice from challenge experiments after lung lavage. Expression was normalized by using the housekeeper gene *peptidyl prolyl isomerase A* (*PPIA*) and is presented as fold difference to the PBS control group. (a, c, g) Data are presented as mean + SEM of *n* = 10–12 animals from *n* = 3 separate experiments. (d–f) Data are generated from *n* = 8–10 mice from two independent experiments. Statistical significance was assessed using a two-tailed Mann-Whitney test and is indicated by ^∗^*p* ≤ 0.05 and ^∗∗^*p* ≤ 0.01.

To further delineate the direct effects of *Ts*-Chit treatment on lung inflammation of OVA-sensitized and OVA-challenged mice, we investigated lung tissue pathology and recruitment of alternatively activated macrophages (AAM). While histopathological findings remained overall similar in the lungs of untreated (OVA) and *Ts*-Chit-treated allergic animals (Figure 5(a)), immunohistochemistry revealed increased numbers of interstitial RELM*α*^+^ cells, a signature marker for AAM that mediates lung vascularization and tissue repair [57, 58] when mice were treated with *Ts*-Chit compared to OVA (Figures 5(b) and 5(c)). A similar trend was observed for inactivated *Ts*-Chit-treated animals. Since this was in contrast to our previous observation on *Ts*-ES L1-induced suppression of RELM*α*^+^ cells [34] and given that the resistin-like molecule (RELM) *α* is expressed not only in macrophages but also in epithelial cells and tissue-infiltrating eosinophils, we used smaller groups of mice to define RELM*α* expression to a interstitial macrophage phenotype by flow cytometry. Lung tissue macrophages were analyzed in homogenates of lavaged lungs and defined as EpCAM^−^CD11c^+^CD64^+^MHCII^+^SiglecF^−^ (Figure 5(d)). While overall frequencies of tissue macrophages were not affected by *Ts*-Chit treatment (Figure 5(e)), we again detected an increase in RELM*α*^+^ macrophages but did not reach statistical significance (Figure 5(f)). However, we found no indication for direct effects of *Ts-*Chit on RELM*α*^+^ expression in macrophages when analyzing RELM*α*^+^ mRNA levels in bone marrow-derived macrophages stimulated with recombinant *Ts-*Chit *in vitro* (data not shown).

Contrary to the increase of RELM*α*^+^ cells in the lung, RT-PCR of homogenized lung tissue of all groups of mice revealed downregulation of *arginase-1* and *MCP-1* mRNA when mice were treated with intact and also inactivated *Ts*-Chit compared to PBS (Figure 5(g)). Supporting reduced numbers of eosinophil infiltration upon *Ts*-Chit treatment, we detected downregulation of CCL11/eotaxin mRNA, a potent eosinophil chemoattractant that stimulates recruitment of eosinophils from the blood to sites of allergic inflammation (Figure 5(e)). Together, these data illustrate that protein integrity of the *Ts*-Chit is only partially responsible for the treatment effects leading to attenuated airway hyperreactivity.

### 3.5. *T. suis* Chitinase Treatment Interferes with Murine Chitinase in AHR

**Figure 6:**
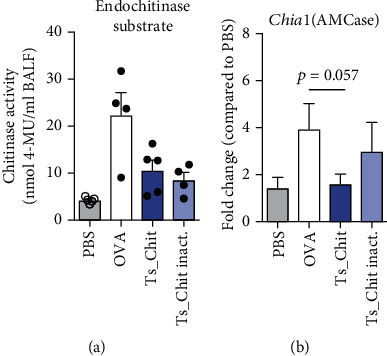
*Ts*-Chit treatment interferes with murine chitinase activity. (a) Chitinase assay testing BAL samples of allergic mice with endochitinase substrate. Data shown are mean + SEM of *n* = 4–5 replicates. (b) mRNA expression of murine chitinase AMCase (*Chia1*) in lung tissue samples of mice from AHR challenge experiments after lung lavage. Expression was normalized by using the housekeeper gene *peptidyl prolyl isomerase A* (*PPIA*) and is presented as fold difference to the PBS group. Data are presented as mean + SEM of *n* = 12 animals from *n* = 3 separate experiments. Data were analyzed statistically using an unpaired *t*-test.

From our *in vitro* and AHR experiments, we concluded that a direct immunomodulatory effect of *T. suis* chitinase or its chitinase activity was very unlikely. However, considering the high sequence similarities to known chitinases and CLPs, we asked whether *Ts*-Chit treatment of allergic mice interfered with the effector functions of its murine counterpart, the acidic mammalian chitinase (AMCase). AMCase is secreted by macrophages and epithelial cells of the lung and gut and in the lungs constitutively expressed and secreted into the airway lumen. STAT6-activating signals, such as IL-13, further induce AMCase expression, and elevated AMCase mRNA and protein levels are detected in BAL of OVA-induced asthmatic mice [59]. Experimentally, the administration of anti-AMCase sera was shown to reduce BAL and tissue eosinophilia and BAL chitinase activity [59]. We therefore examined BAL fluid of allergic mice for chitinase activity. And indeed, chitinase activity was found to be reduced in the BAL of allergic mice treated with intact and inactivated *Ts*-Chit compared to untreated (OVA) mice (Figure 6(a)). In line with that, we detected decreased AMCase transcript level (*Chia1*) mRNA transcripts in lung tissue snips of allergic *Ts*-Chit-treated compared to untreated (OVA) mice (Figure 6(b)). We therefore examined the implications of *Ts*-Chit treatment on the family of murine chitinases and chitinase-like proteins (CLP) that are highly implicated in pathology of Th2-mediated allergic airway inflammation [60]. Interestingly, *T. suis* chitinase was shown to have a high percentage of amino acid sequence identity when compared to the two true murine chitinases (AMCase (41.6%) and Chit1 (41.5%)) or the murine CLPs Ym1 (39%), Ym2 (37.5%), or BRP-39 (36.7%) (Suppl. Fig. 2); its protein structure, however, is not known so far.

### 3.6. *T. suis* Chitinase Reveals High Structural Similarity to Murine Chitinase but Presents Dimeric Structure

**Figure 7:**
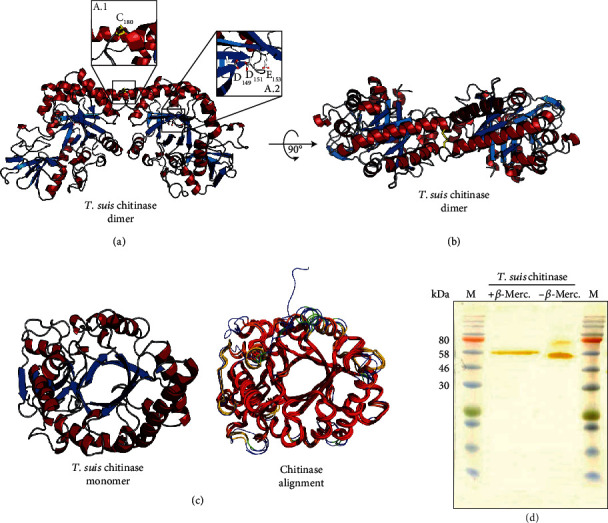
Three-dimensional structure of *T. suis* chitinase (*Ts-*Chit). (a) Overall structure of *Ts*-Chit forming a C2-symmetric dimer (*α*-helices in red, ß-strands in blue). The inset A.1 depicts the dimer-stabilizing disulfide bond-forming cysteine residue at position 180, while the inset A.2 highlights the catalytic DxDxE center. (b) *Ts*-Chit dimer rotated by 90° compared to (a). (c) *Ts*-Chit monomer and its structural alignment with AMCase (PDB Id 3FXY, [84]), human Chit1 (PDB-Id 1GUV, [85]), human YKL-40 (PDB-Id 1NWR, [86]), and murine Ym1 (PDB-Id 1VF8, [87]). (d) Silver-stained SDS/PAGE gel of recombinantly expressed *Ts*-Chit (1 *μ*g) under reducing (+ß-Merc.) and nonreducing (-ß-Merc.) loading conditions.

To better understand structural differences between nematode and mammalian chitinases, we determined the crystal structure of the nematode chitinase protein by X-ray crystallography. The final model analysis data collection and refinement statistics are summarized in Table 3. They indicate that the structure is of high resolution, that it is well refined to very good statistics, and hence that it is of high quality. The overall structure reveals a TIM-barrel or (*βα*)8-barrel motif, which means that 8 *β*-strands form the central barrel which is surrounded by 8 *α*-helices (Figures 7(a) and 7(c)). The active site with the sequence motif D_149_xD_151_xE_153_ is depicted in Figure 7 A.2. The crystal structure furthermore revealed a C2-symmetric dimer of the protein, which is stabilized by an intermolecular disulfide bond formed by a cysteine residue at position 180 (Figures 7 A.1 and 7(b)). Such dimerization has not been described so far for human or murine chitinases, nor has the occurrence of a cysteine residue at position 180. In addition, the structural alignment with human AMCase (3FXY), human chitotriosidase (1GUV), human YKL-40 (1NWR), and murine Ym1 (1VF8) (Figure 7(c)) indicates highly similar structural features in the core regions (marked in red) and certain areas, mostly at the protein surface, where the structure differs between nematode and mammalian chitinases (highlighted in green/blue). A detailed structure-based sequence alignment of *T. suis* chitinase, mouse chitinases, and CLPs is given in Supplementary S2. Notably, the C-terminal region of *Ts*-Chit from amino acids 401 to 495 contains many threonine repeats and is referred to as unordered region. The *T. suis* chitinase dimerization was further examined by comparing reducing (+ *β*-mercaptoethanol) and nonreducing (- *β*-mercaptoethanol) conditions for SDS/PAGE loading. We indeed detected a second, higher band at around 75 kDa indicative for dimerization under nonreducing conditions (Figure 7(d)).

**Figure 8:**
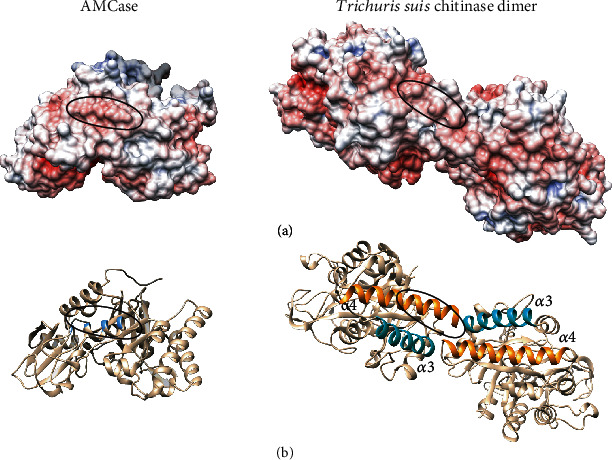
Electrostatic surface representation of mammalian AMCase (PDB 3FY1) and the *Ts*-Chit dimer. (a) In the *Ts*-Chit dimer, a surface patch of similar charge and shape as observed in mammalian AMCase is formed. The similar, acidic surface patch in both molecules is circled. (b) Cartoon rendering of AMCase and *Ts*-Chit in the same orientation as in the upper panel. The location of the circled residues in AMCase is highlighted in blue. Helices *α*3 and *α*4 contributing to the dimer formation in *Ts*-Chit are colored turquoise and orange, respectively. The surface electrostatics were calculated with APBS and visualized with UCSF Chimera.

Following the idea that *T. suis* interferes with murine chitinase activity during allergic airway hyperreactivity, we more specifically compared surface features of *Ts*-Chit and AMCase (PDB: 3FY1), the only acidic chitinase with known experimental 3D structure. To that effect, the surface electrostatics for mammalian AMCase and the *Ts*-Chit monomer were visualized and appraised. While investigating the monomer alone did not reveal any strikingly identical surface patches, looking at the dimerization region around *α*3/*α*4-*α*3′/*α*4′ (*α*3:~127-142; *α*4:~161-180) revealed similarities with an acidic surface patch of AMCase (Figure 8(a)).

In summary, this first crystallographic structure analysis of a parasitic nematode chitinase revealed *Ts*-Chit dimerization and the concomitant existence of surface patches absent in the monomeric protein. The surface electrostatics of these dimer-specific patches show a remarkable similarity between *T. suis* chitinase and murine AMCase. The structural und functional similarity of *T. suis* chitinase and murine AMCase could either mean that *Ts*-Chit is sensed similar to murine AMCase resulting in cumulative effects or in turn that *Ts*-Chit interferes with murine AMCase function during lung inflammation.

## 4. Discussion

An efficient strategy of parasitic nematodes to ensure survival, growth, and reproduction in immunocompetent hosts involves immunoregulation of the host immune response. Being equipped with excretory and secretory systems [61], parasitic nematodes release several products (ES products) that shape their host immune response [1]. It is those yet unidentified ES immunomodulatory molecules that bear the therapeutic potential for targeting immune-mediated diseases.

Following the complex life cycles of parasitic nematodes, there is evidence for life cycle stage-specific expression and release of distinct sets of ES molecules needed to fulfill specific tasks [31, 62, 63]. We have previously reported that total ES proteins of the very early larval stage (L1) of the whipworm *T. suis* very efficiently ameliorate allergic lung disease in a model of OVA-induced AHR when applied during the sensitization phase [34]. Notably, in TSO therapy for indications such as ulcerative colitis, Crohn's disease, or various autoimmune diseases including diabetes and multiple sclerosis (MS), patients are repeatedly exposed to the early larval stages of *T. suis* owing to the fact that *T. suis* only transiently infects human intestines [23, 64]. Patients receiving TSO therapy, however, seem to not equally benefit from the worm therapy [22, 26–28]. In this context, we observed a considerable degree of interindividual variability in the adaptive immune responses to this helminth treatment [29] suggesting that even how the anti-TSO response is formed depends on the environmental and inflammatory context of the individual treatment.

This led us to suggest a role for mediators being released very early during infection by the first larval stages, and we therefore focused on the identification of specific L1 ES immunomodulators of *T. suis* and their potential immunomodulatory mechanisms. We compared ES proteins collected from hatched L1 versus later larval stages (10, 18, and 28 day old) isolated from the intestines of infected pigs by using LC-MS/MS. Among the 33 proteins that were identified in the ES of *in vitro*-hatched *T. suis* L1, 8 proteins were also found in the ES of L2 larvae, but no overlap to later larval stages. We concentrated on proteins selectively expressed by the first two larval stages (L1 and L2), equipped with a signal peptide and devoid of a transmembrane domain. A large proportion of proteins detected in *T. suis* ES are not encoded with a signal peptide, which is in line with reports from other species [65–67]. Over the course of this study, the genome of the porcine whipworm *T. suis* published in 2014 [68] had been complemented with two slightly larger assemblies of male and female worms that in turn added on to the number of identified protein IDs.

Screening six recombinantly expressed L1 ES proteins, eukaryotically produced in *L. tarentolae*, in a model of OVA-induced allergic airway hyperreactivity revealed that the application of *T. suis* KFD48490.1 markedly reduced inflammatory lung infiltrates, partly mimicking the effects of total *T. suis* L1 ES [34]. Our results further proved that KFD48490.1 is an enzymatically active chitinase of *T. suis*. The enzyme retained its high activity in the pH range from 4 to 6 and thus is relatively resistant to acidic pH similar to what has been documented for acidic mammalian chitinases [69, 70].

Chitinases are glycosyl hydrolases (GH) with the ability to directly degrade chitin polymers to low molecular weight chitooligomers (reviewed in [71]). Based on amino acid sequence similarity, the *T. suis* chitinase belongs to GH family 18 characterized by an enzyme core of 8 *β*-strands forming a barrel that is surrounded by *α*-helices, as well as the conserved sequence DxDxE forming the active site. Chitinases have essential roles in the life cycle of many parasites, supported by the existence of multiple genes as well as tissue- and stage-specific expression patterns [72–74]. Chitinase activity in helminth biology is thought to be essential during embryonic development, larval molting, and degradation of the chitinous matrix [75, 76], suggesting that chitinases might be attractive intervention targets [50].

In the context of allergic lung disease, however, chitinases play a key role in mediating the Th2-driven inflammatory responses commonly associated with asthma and have been investigated to indicate disease severity [59, 77, 78]. Mice express two enzymatically active chitinases, acidic mammalian chitinase (AMCase) and chitotriosidase (Chit), and a set of enzymatically inactive chitinase-like proteins (Ym1, Ym2, and BRP39). AMCase is secreted by macrophages and epithelial cells of the lung and gut. In the lungs, it is constitutively expressed and secreted into the airway lumen. STAT6-activating signals, such as IL-13, further induce AMCase expression, and elevated AMCase mRNA and protein levels are detected in BAL of OVA-induced asthmatic mice [59]. Even though the mechanism of action of most mammalian chitinases and CLPs is not fully understood, stimulation of alveolar macrophages and enhancing tissue inflammation are likely to be involved. Hartl and coworkers [79] who showed that AMCase physically interacts with epidermal growth factor receptor (EGFR) to induce the production of, e.g., MCP-1, CCL17, and IL-8, in lung epithelial cells also reported an autocrine and/or paracrine feedback mechanism for AMCase in protecting airway epithelial cells from Fas ligand- (FasL-) and growth factor withdrawal-induced apoptosis [79, 80]. Therefore, it is not surprising that blocking AMCase activity by administration of anti-AMCase sera reduced BAL and tissue eosinophilia [59]. Similarly, in a gut inflammation model, it has been shown that the panchitinase inhibitor caffeine was able to dampen inflammation in a DSS-induced colitis model [81].

We now demonstrate that treating mice with a worm-derived chitinase from *T. suis* (*Ts*-Chit) during sensitization reduced BAL infiltrates and eosinophilia, increased numbers of RELM*α*^+^ interstitial cells, and improved overall lung function. Finding increased RELM*α*^+^ lung macrophages might reflect an increased capacity of *Ts-*Chit-treated animals to limit Th2-mediated inflammation and initiate tissue repair [57].

Disease amelioration was linked to enhanced local Th2 responses in lung tissues, and heat-inactivated *Ts*-Chit was able to partly phenocopy these effects *in vivo*, suggesting that the functional chitinase domain is not entirely responsible for the effects observed. Using different *in vitro* assays, we investigated potential immunomodulatory effects of *Ts-*Chit on DC and T cell activation but found no evidence for a direct effect on immune cells as shown in other studies [31, 55]. Yet, we cannot rule out any direct cellular effects of *Ts*-Chit on mammalian cells.

Our *in vivo* results, however, suggest a mechanism related to the one suggested by Zhu and colleagues [59] that report on blocking AMCase activity using antiserum treatment to reduce AHR severity. Similar to that study, we show here that *Ts*-Chit treatment had no suppressive effects on Th2 inductive responses such IL-4 and IL-13 as well as IL-5 levels. Zhu and coworkers further demonstrated that anti-AMCase sera diminished the ability of IL-13 to stimulate eotaxin and monocyte-chemotactic protein 1 (MCP-1), reflected by our results following treatment with the worm chitinase. Besides anti-AMCase treatment, other approaches have been used to address AMCase activity, such as inhibition with small molecule [82] or RNA interference [83], and demonstrated suppressed eotaxin, MCP-1, and MIP-1ß levels when AMCase activity or expression was inhibited.

Sequence alignment demonstrates a relatively high degree of similarity between *T. suis* chitinase and AMCase, but also to CLPs such as YM1, Ym2, or BRP39. Given the high homology of *T. suis* and murine chitinases, both summative and interfering effects would be conceivable. Our *in vivo* observations on reduced chemokine expression and eosinophil infiltration, but nonsuppressed Th2 responses, rather points towards a scenario where treatment with the worm chitinase interferes with the host's own chitinase activity triggered upon lung inflammation. As a first indication, we observed decreased host chitinase activity in BAL and a decrease in lung tissue AMCase transcripts following treatment with the structurally highly similar worm chitinase. Whether these findings are a consequence of the overall reduced lung inflammation or result from chitinase protein interference still needs to be verified.

The X-ray crystal structure of *Ts*-Chit reported here confirmed the expected high structural similarity of the nematode chitinase with murine acidic mammalian chitinase. In addition, analysis of surface electrostatics of the *Ts*-Chit dimer suggests the existence of an acidic surface patch on the nematode protein that bears similarity to a surface feature on AMCase monomers. This might point to molecular mimicry mechanisms, a phenomenon that is triggered by a high degree of similarity of pathogen and host epitopes. Future studies need to investigate whether structural similarity between *T. suis* chitinase and murine chitinases or CLPs is potentially linked to a serological response limiting activity of host AMCase similar to the study of Zhu and colleagues that applied anti-AMCase sera to ameliorate lung inflammation [59].

## 5. Conclusion

In summary, we investigated L1 stage-specific, secreted proteins of *T. suis* and their capacity for immunomodulation of allergic lung disease. We identified an active chitinase of *T. suis* that, when applied during sensitization of an allergic response, reduced eosinophilia and lung chemokine responses. These findings add to our understanding of specific functions of early secreted *T. suis* products in helminth biology and when considered as therapeutics to create an anti-inflammatory environment. The structural similarity between the worm chitinase and its host equivalent, the absence of direct cellular effects, and the potential to interfere with murine chitinase activity suggest an alternative mechanism of *Ts*-Chit ameliorating OVA-induced, allergic lung disease that likely involves structural mimicry. Such a mechanism of immunomodulation could be one of those unrecognized strategies of mediating anti-inflammatory responses.

## Figures and Tables

**Table 1:** Sequences of oligonucleotides.

Gene	Fwd primer (5′-3′)	Rev primer (5′-3′)
*Chia1* (AMCase)	CCCTTGGCATATCCACTGA	ACAGAATCCACTGCCTCCAG
*Ccl11* (eotaxin1)	CTGCTTGATTCCTTCTCTTTCCTAA	GGAACTACATGAAGCCAAGTCCTT
*Ccl2* (MCP-1)	TTAAAAACCTGGATCGGAACCAA	GCATTAGCTTCAGATTTACGGGT
*Arg1* (arginase)	CAGAAGAATGGAAGAGTCAG	CAGATATGCAGGGAGTCACC
Housekeeping gene *PPIA*	GAGCTGTTTGCAGACAAAGTTC	CCCTGGCACATGAATCCTGG

**Table 2:** MALDI-TOF MS verified T. suis L1 ES proteins that were selected and recombinantly expressed using LEXSY.

NCBI-ID	MW (kDa)	Known homology	N-Glycosylation	O-Glycosylation
KFD48490.1	55.45	Acidic mammalian chitinase (*T. trichiura*)	—	28
KFD55729.1	13.89	—	—	1
KFD54802.1	31.06	CAP domain-containing protein (*T. trichiura*)	3	10
KFD45500.1	37.04	Venom allergen 5 (*T. trichiura*)	1	24
KFD47618.1	14.18	Hypothetical protein (*T. trichiura*)	—	—
KFD53667.1^∗^	31.06	Hypothetical protein (*T. trichiura*)	5	—

Sequence similarity search for protein homology was performed using BLAST/NCBI. Glycosylation prediction was performed using NetOGlyc 4.0.0.13 for O-glycosylation and NetNGlyc 1.0 Server for N-glycosylation. ^∗^Formerly identified using HelmDB database and recombinantly expressed based on this sequence.

**Table 3:** X-ray-based structure analysis data collection and refinement statistics.

Crystallographic data	*Ts*-Chit
Space group	C2
Cell parameters	*a*, *b*, *c* (Å) 196, 52.6, 93.5 *α*, *β*, *γ* (°) 90, 108.8, 90
Wavelength (Å)	0.91841
Resolution (Å)	47.9-1.65
Total reflections	609875
Unique reflections	209810
Completeness (%)	98.3 (97.4)
CC1/2	0.99 (0.32)
*I*/*σ* (I)	7.42 (0.31)
*Refinement statistics*	
No. of reflections used	209322
No. of reflections in test set	2200
*R* factor	0.191
*R*_free_	0.226
RMS deviation	
Bonds (Å)	0.007
Angles (°)	0.863
Mean *B* factor (Å^2^)	44.11
*Ramachandran plot*	
Residues in most favored regions	98.91
Residues in additional allowed regions	1.09

## Data Availability

The authors provide the MS/MS dataset in the Supplementary Information files. Additional data will be made available through the authors upon request.

## Abbreviations

AAM: Alternatively activated macrophages
AHR: Airway hyperreactivity
AMCase: Acidic mammalian chitinase
BAL: Bronchoalveolar lavage
Chit: Chitinase
CLPs: Chitinase-like proteins
ES: Excretory-secretory
ESP: Excretory-secretory proteins
GH: Glycosyl hydrolases
L1: Fist larval stage
LEXSY: Leishmania expression system
MS: Mass spectrometry
OVA: Ovalbumin
RELM*α*: Resistin-like molecule alpha
SPs: Soluble proteins
TCEP: Tris(2-carboxyethyl)phosphine
*T. suis*: *Trichuris suis*
*Ts*-Chit: *Trichuris suis* chitinase
*Ts*-ES: *Trichuris suis* excretory-secretory proteins
TSO: *Trichuris suis* ova
TTO: *Trichuris trichiura* ova
UC: Ulcerative colitis.

## Supplementary Materials

Supplementary Materials Table S1: mass spectrometric identification of *T. suis* larval ES proteins. Precipitated proteins were reduced, alkylated, tryptic digested, and analyzed by LC-MSMS and Mascot searches against the NCBI database. Significant hits (+) were identified with >2 peptides (*p* < 0.01); nonsignificant candidates (c) are also listed. Homologous proteins are grouped to the same number. S2: comparison of topology and secondary structure elements of *T. suis* chitinase with mouse chitinases and chitinase-like proteins (CLPs). Structure-based amino acid sequence alignment of *T. suis* chitinase (*Ts*-Chit) with murine Ym1 (*Chil3*), Ym2 (*Chil4*), chitotriosidase isoforms 1 and 2 (*Chit1_1*, *Chit1_2*), AMCase (*Chia*), and BRP-39 (*Chil1*). Secondary structure elements were predicted and visualized with PSIPRED [1] and SBAL [2], indicating *α*-helices in green, *β*-strands in red, and cysteines in yellow. The top line shows the annotation with secondary structure elements observed in the crystal structure of *Ts*-Chit and the catalytic motive DxDxE [44, 45].
