# Supplementary material for: A Helminth-Derived Chitinase Structurally Similar to Mammalian Chitinase Displays Immunomodulatory Properties in Inflammatory Lung Disease
Source: J Immunol Res. 2021 Nov 25;2021:6234836. doi: 10.1155/2021/6234836 (PMC8639245; doi:10.1155/2021/6234836)
Supplement: Supplementary Materials — Table S1: mass spectrometric identification of T. suis larval ES proteins. Precipitated proteins were reduced, alkylated, tryptic digested, and analyzed by LC-MSMS and Mascot searches against the NCBI database. Significant hits (+) were identified with >2 peptides (p < 0.01); nonsignificant candidates (c) are also listed. Homologous proteins are grouped to the same number. S2: comparison of topology and secondary structure elements of T. suis chitinase with mouse chitinases and chitinase-like proteins (CLPs). Structure-based amino acid sequence alignment of T. suis chitinase (Ts-Chit) with murine Ym1 (Chil3), Ym2 (Chil4), chitotriosidase isoforms 1 and 2 (Chit1_1, Chit1_2), AMCase (Chia), and BRP-39 (Chil1). Secondary structure elements were predicted and visualized with PSIPRED [1] and SBAL [2], indicating α-helices in green, β-strands in red, and cysteines in yellow. The top line shows the annotation with secondary structure elements observed in the crystal structure of Ts-Chit and the catalytic motive DxDxE [44, 45]. [file 6234836.f1.zip › Supplementary Data S2.docx]

**Supporting information to:**

**A helminth-derived chitinase structurally similar to mammalian chitinase displays immunomodulatory properties in inflammatory lung disease.**

Friederike Ebner^1*^, Katja Lindner^1*^, Katharina Janek^2^, Agathe Niewienda^2^, Piotr H. Malecki^3,4^, Manfred S. Weiss^3^, Tara E. Sutherland^5^, Arnd Heuser^6^, Anja A. Kühl^7^, Jürgen Zentek^8^, Andreas Hofmann^9^, and Susanne Hartmann^1^

^1^Institute of Immunology, Department of Veterinary Medicine, Freie Universität Berlin, Berlin, Germany, ^*^equal contribution

^2^Charité – Universitätsmedizin Berlin, corporate member of Freie Universität Berlin, Humboldt-Universität zu Berlin, and Berlin Institute of Health, Institute of Biochemistry, Shared Facility for Mass Spectrometry, Berlin, Germany

^3^Macromolecular crystallography (HZB-MX), Helmholtz-Zentrum Berlin, Berlin, Germany

^4^current address: International Institute of Molecular and Cell Biology in Warsaw (IIMCB), Poland

^5^Lydia Becker Institute of Immunology and Inflammation, Faculty of Biology, Medicine and Health, Manchester Academic Health Science Centre, University of Manchester, Manchester, United Kingdom

^6^Max Delbrück Center for Molecular Medicine (MDC), Pathophysiology Platform, Berlin, Germany

^7^Charité *–* Universitätsmedizin Berlin, corporate member of Freie Universität Berlin and Humboldt-Universität zu Berlin, iPATH.Berlin, Berlin, Germany

^8^Institute of Animal Nutrition, Department of Veterinary Medicine, Freie Universität Berlin, Berlin, Germany

^9^Department of Veterinary Biosciences, Melbourne Veterinary School, The University of Melbourne, Parkville, Victoria 3010, Australia

**Corresponding author:**

Prof. Dr. Susanne Hartmann

Freie Universität Berlin, Department of Veterinary Medicine, Institute of Immunology

T: +49 30 838-51824, F: +49 30 838-451834

E: [susanne.hartmann@fu-berlin.de](mailto:susanne.hartmann@fu-berlin.de)

**Corresponding author:**

Prof. Dr. Susanne Hartmann

T: +49 30 838-51824, F: +49 30 838-451834

E: susanne.hartmann@fu-berlin.de

**Supplementary Information S2**

**
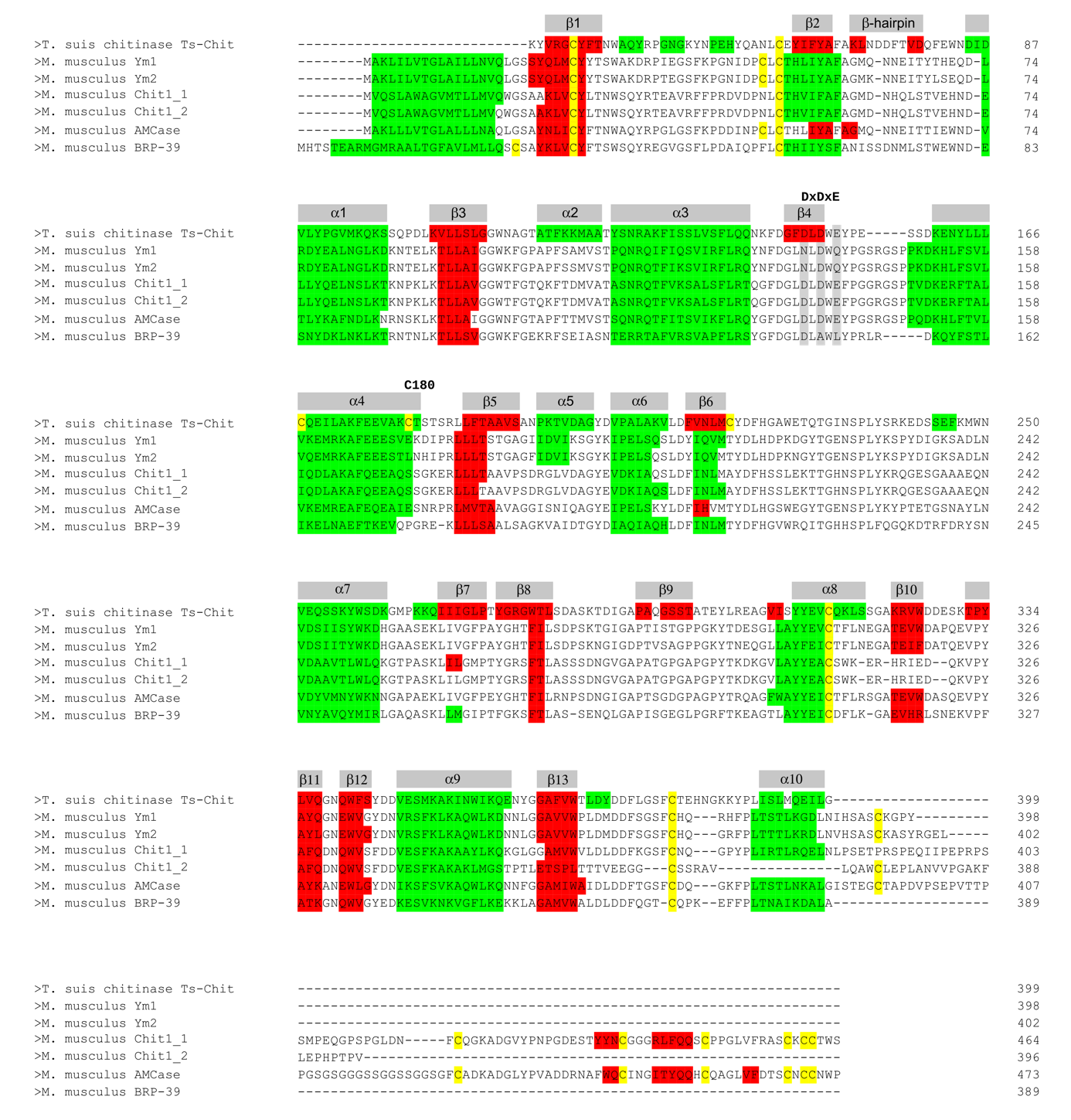
 S2. Comparison of topology and secondary structure elements of *T. suis* chitinase with mouse chitinases and chitinase like proteins (CLPs).** Structure-based amino acid sequence alignment of *T. suis* chitinase (*Ts*-Chit) with murine Ym1 (*Chil3*), Ym2 (*Chil4*), chitotriosidase isoforms 1 & 2 (*Chit1_1, Chit1_2*), AMCase (*Chia*) and BRP-39 (*Chil1*). Secondary structure elements were predicted and visualized with PSIPRED [1] and SBAL [2], indicating α-helices in green, β-strands in red and cysteines in yellow. The top line shows the annotation with secondary structure elements observed in the crystal structure of *Ts*-Chit and the catalytic motive DxDxE.

**References**

1. Bryson K, McGuffin LJ, Marsden RL, Ward JJ, Sodhi JS, Jones DT. Protein structure prediction servers at University College London. Nucleic Acids Res. 2005;33: W36–W38.

2. Wang CK, Broder U, Weeratunga SK, Gasser RB, Loukas A, Hofmann A. SBAL: a practical tool to generate and edit structure-based amino acid sequence alignments. Bioinformatics. 2012;28: 1026–1027. doi:10.1093/bioinformatics/bts035
