# Supplementary material for: A Helminth-Derived Chitinase Structurally Similar to Mammalian Chitinase Displays Immunomodulatory Properties in Inflammatory Lung Disease
Source: J Immunol Res. 2021 Nov 25;2021:6234836. doi: 10.1155/2021/6234836 (PMC8639245; doi:10.1155/2021/6234836)
Supplement: Supplementary Materials — Table S1: mass spectrometric identification of T. suis larval ES proteins. Precipitated proteins were reduced, alkylated, tryptic digested, and analyzed by LC-MSMS and Mascot searches against the NCBI database. Significant hits (+) were identified with >2 peptides (p < 0.01); nonsignificant candidates (c) are also listed. Homologous proteins are grouped to the same number. S2: comparison of topology and secondary structure elements of T. suis chitinase with mouse chitinases and chitinase-like proteins (CLPs). Structure-based amino acid sequence alignment of T. suis chitinase (Ts-Chit) with murine Ym1 (Chil3), Ym2 (Chil4), chitotriosidase isoforms 1 and 2 (Chit1_1, Chit1_2), AMCase (Chia), and BRP-39 (Chil1). Secondary structure elements were predicted and visualized with PSIPRED [1] and SBAL [2], indicating α-helices in green, β-strands in red, and cysteines in yellow. The top line shows the annotation with secondary structure elements observed in the crystal structure of Ts-Chit and the catalytic motive DxDxE [44, 45]. [file 6234836.f1.zip › Supplementary Table S1.docx]

|  |  |  |  |  |  |  |
| --- | --- | --- | --- | --- | --- | --- |
| **Table S1:** Mass spectrometric identification of *T. suis* larvae ES proteins. | |  |  |  |  |  |
| Precipitated proteins were reduced, alkylated, tryptic digested and analyzed by LC-MSMS and Mascot searches against NCBI database. | | | | | | |
| Significant hits (+) were identified with > 2 peptides (p< 0.01), non-significant candidates (c) are also listed. | | | |  |  |  |
| Homologous proteins are grouped to the same #-number. | |  |  |  |  |  |
|  |  |  |  |  |  |  |
| **#** | **Protein name** | **NCBI-ID** | **L1** | **10dpi** | **18dpi** | **28dpi** |
| 1_1 | astacin [Trichuris suis] | KHJ43437.1 | **+** |  |  |  |
| 1_2 | hypothetical protein M513_10127 [Trichuris suis] | KFD48975.1 | **+** |  |  |  |
| 2_1 | hypothetical protein D918_10164 [Trichuris suis] | KHJ39810.1 | **+** |  |  |  |
| 2_2 | hypothetical protein M513_00865 [Trichuris suis] | KFD58102.1 | **+** |  |  |  |
| 2_3 | hypothetical protein M514_00865 [Trichuris suis] | KFD61181.1 | **+** |  |  |  |
| 3_1 | hypothetical protein M513_06373 [Trichuris suis] | KFD52717.1 | **+** | **c** |  |  |
| 3_2 | serine proteinase inhibitor [Trichuris suis] | KHJ48406.1 | **+** | **c** |  |  |
| 4_1 | chitin binding Peritrophin-A domain protein [Trichuris suis] | KHJ46953.1 | **+** |  |  |  |
| 4_2 | hypothetical protein M513_10624 [Trichuris suis] | KFD48490.1 | **+** |  |  |  |
| 4_3 | hypothetical protein M514_10624, partial [Trichuris suis] | KFD69702.1 | **+** |  |  |  |
| 5_1 | hypothetical protein D918_01317 [Trichuris suis] | KHJ48050.1 | **+** | **c** |  |  |
| 5_2 | hypothetical protein M513_03477 [Trichuris suis] | KFD55729.1 | **+** | **c** |  |  |
| 6_1 | hypothetical protein M513_01545 [Trichuris suis] | KFD57442.1 | **+** | **+** |  |  |
| 6_2 | hypothetical protein M514_01545 [Trichuris suis] | KFD66550.1 | **+** | **+** |  |  |
| 7_1 | chitinase class I [Trichuris suis] | KHJ45348.1 | **+** |  |  |  |
| 7_2 | hypothetical protein M513_11522 [Trichuris suis] | KFD47603.1 | **+** |  |  |  |
| 7_3 | hypothetical protein M514_11522 [Trichuris suis] | KFD67631.1 | **+** |  |  |  |
| 8_1 | hypothetical protein D918_02479 [Trichuris suis] | KHJ46940.1 | **+** |  |  |  |
| 8_3 | hypothetical protein M513_13299 [Trichuris suis] | KFD45818.1 | **+** |  |  |  |
| 9_1 | copper/zinc superoxide dismutase [Trichuris suis] | KHJ41357.1 | **+** |  |  |  |
| 9_2 | hypothetical protein M513_05621 [Trichuris suis] | KFD53515.1 | **+** |  |  |  |
| 9_3 | hypothetical protein M514_05621 [Trichuris suis] | KFD71814.1 | **+** |  |  |  |
| 10_1 | hypothetical protein M513_11481 [Trichuris suis] | KFD47618.1 | **+** |  |  |  |
| 10_2 | hypothetical protein M514_11481 [Trichuris suis] | KFD72328.1 | **+** |  |  |  |
| 11 | hypothetical protein M513_05526 [Trichuris suis] | KFD53610.1 | **+** | **+** |  |  |
| 12 | hypothetical protein M513_01533 [Trichuris suis] | KFD57430.1 | **+** | **+** |  |  |
| 13 | hypothetical protein M513_07350, partial [Trichuris suis] | KFD51823.1 | **+** | **+** |  |  |
| 14 | hypothetical protein M513_00025 [Trichuris suis] | KFD58862.1 | **+** | **+** |  |  |
| 15 | hypothetical protein M513_05372 [Trichuris suis] | KFD53667.1 | **+** | **c** |  |  |
| 16 | hypothetical protein M513_05523, partial [Trichuris suis] | KFD53607.1 | **+** |  |  |  |
| 17 | hypothetical protein M513_04236 [Trichuris suis] | KFD54802.1 | **+** |  |  |  |
| 18 | hypothetical protein M513_13623 [Trichuris suis] | KFD45500.1 | **+** |  |  |  |
| 19 | hypothetical protein M513_04624 [Trichuris suis] | KFD54477.1 | **+** |  |  |  |
| 20 | hypothetical protein M513_09085 [Trichuris suis] | KFD49992.1 | **c** | **+** | **+** | **+** |
| 21 | hypothetical protein M513_04701 [Trichuris suis] | KFD54358.1 | **c** | **+** | **+** | **+** |
| 22 | hypothetical protein M513_06879 [Trichuris suis] | KFD52316.1 | **c** | **+** |  |  |
| 23_1 | hypothetical protein M514_10152 [Trichuris suis] | KFD62925.1 | **c** | **c** | **+** | **+** |
| 23_2 | hypothetical protein M513_10152 [Trichuris suis] | KFD49000.1 | **c** | **c** |  |  |
| 24_1 | hypothetical protein M513_01669 [Trichuris suis] | KFD57566.1 | **c** | **c** |  |  |
| 24_2 | hypothetical protein M514_01669 [Trichuris suis] | KFD64855.1 | **c** | **c** |  |  |
| 25_1 | hypothetical protein M514_24957 [Trichuris suis] | KFD62836.1 | **c** | **c** |  |  |
| 25_2 | hypothetical protein M513_03628 [Trichuris suis] | KFD55576.1 |  | **c** |  |  |
| 25_3 | hypothetical protein M513_03629 [Trichuris suis] | KFD55577.1 |  | **c** |  |  |
| 25_4 | hypothetical protein M514_03629, partial [Trichuris suis] | KFD59516.1 |  | **c** |  |  |
| 26 | hypothetical protein D918_04545 [Trichuris suis] | KHJ45241.1 | **c** | **c** |  |  |
| 27 | hypothetical protein M513_01541 [Trichuris suis] | KFD57438.1 | **c** | **c** |  |  |
| 28 | hypothetical protein M513_00679 [Trichuris suis] | KFD58453.1 | **c** |  |  |  |
| 29_1 | hypothetical protein M513_11174 [Trichuris suis] | KFD47941.1 |  | **+** | **+** | **+** |
| 29_2 | hypothetical protein M513_11839 [Trichuris suis] | KFD47286.1 |  | **+** | **+** | **+** |
| 29_3 | hypothetical protein M514_23670 [Trichuris suis] | KFD64181.1 |  | **+** | **+** | **c** |
| 29_4 | hypothetical protein M514_27543 [Trichuris suis] | KFD60271.1 |  | **+** | **+** | **+** |
| 30_1 | hypothetical protein M513_03282 [Trichuris suis] | KFD55843.1 |  | **+** | **+** | **+** |
| 30_2 | hypothetical protein M514_03282 [Trichuris suis] | KFD70192.1 |  | **+** | **+** | **+** |
| 30_3 | kinase domain protein [Trichuris suis] | KHJ49431.1 |  |  | **+** |  |
| 31_1 | hypothetical protein M513_07694 [Trichuris suis] | KFD51481.1 |  | **+** | **+** | **+** |
| 31_2 | hypothetical protein M514_07694 [Trichuris suis] | KFD61942.1 |  | **+** | **+** | **+** |
| 32_1 | hypothetical protein M513_03048 [Trichuris suis] | KFD55924.1 |  | **+** | **+** | **+** |
| 32_2 | hypothetical protein M514_03048, partial [Trichuris suis] | KFD68317.1 |  | **+** | **+** | **+** |
| 32_3 | trypsin Inhibitor like cysteine rich domain protein [Trichuris suis] | KHJ43166.1 |  | **+** | **+** | **+** |
| 33_1 | hypothetical protein M513_05555 [Trichuris suis] | KFD53639.1 |  | **+** | **c** | **c** |
| 33_2 | hypothetical protein D918_02595 [Trichuris suis] | KHJ47049.1 |  | **c** | **c** | **c** |
| 34_1 | hypothetical protein D918_03253 [Trichuris suis] | KHJ46205.1 |  | **+** |  | **c** |
| 34_2 | hypothetical protein M513_07983 [Trichuris suis] | KFD51083.1 |  | **+** |  | **c** |
| 35_1 | hypothetical protein M514_03300, partial [Trichuris suis] | KFD70210.1 |  | **+** |  | **+** |
| 35_2 | hypothetical protein D918_06438 [Trichuris suis] | KHJ43528.1 |  | **c** |  | **+** |
| 36_1 | hypothetical protein M513_09923 [Trichuris suis] | KFD49201.1 |  | **+** |  | **c** |
| 36_2 | SCP-like protein [Trichuris suis] | KHJ49366.1 |  | **+** |  | **c** |
| 36_3 | hypothetical protein M513_04750 [Trichuris suis] | KFD54407.1 |  |  |  | **c** |
| 37_1 | dipeptidyl carboxydipeptidase family protein [Trichuris suis] | KHJ48157.1 |  | **+** |  | **c** |
| 37_2 | hypothetical protein M513_07445 [Trichuris suis] | KFD51748.1 |  | **+** |  | **c** |
| 37_3 | hypothetical protein M514_07445 [Trichuris suis] | KFD67136.1 |  | **+** |  | **c** |
| 38_1 | hypothetical protein M513_12276 [Trichuris suis] | KFD46830.1 |  | **+** |  | **c** |
| 38_2 | hypothetical protein M514_12276 [Trichuris suis] | KFD64998.1 |  | **+** |  | **c** |
| 39_1 | hypothetical protein M513_01631 [Trichuris suis] | KFD57528.1 |  | **+** | **c** | **c** |
| 39_2 | hypothetical protein M514_01631 [Trichuris suis] | KFD63563.1 |  | **+** | **c** | **c** |
| 40_1 | glycerol-3-phosphate dehydrogenase [NAD(P)+ ] [Trichuris suis] | KHJ43845.1 |  | **+** | **+** | **c** |
| 40_2 | hypothetical protein M513_02985, partial [Trichuris suis] | KFD56207.1 |  | **+** | **+** | **+** |
| 41_1 | hypothetical protein M514_13515 [Trichuris suis] | KFD64188.1 |  | **+** |  | **c** |
| 41_2 | hypothetical protein M513_11170 [Trichuris suis] | KFD47937.1 |  |  |  | **c** |
| 42_1 | hypothetical protein M514_12219 [Trichuris suis] | KFD60943.1 |  | **+** | **+** | **+** |
| 42_2 | hypothetical protein M513_12222 [Trichuris suis] | KFD46900.1 |  | **c** | **+** |  |
| 43_1 | hypothetical protein M514_12825 [Trichuris suis] | KFD62209.1 |  | **+** | **+** |  |
| 43_2 | hypothetical protein M513_12827 [Trichuris suis] | KFD46292.1 |  | **c** | **+** |  |
| 43_3 | hypothetical protein D918_04921 [Trichuris suis] | KHJ44687.1 |  | **c** |  |  |
| 44_1 | hypothetical protein M514_28048 [Trichuris suis] | KFD59771.1 |  | **+** | **c** | **c** |
| 44_2 | hypothetical protein M513_13257, partial [Trichuris suis] | KFD45867.1 |  |  | **c** | **c** |
| 45_1 | hypothetical protein M513_01063 [Trichuris suis] | KFD58300.1 |  | **+** | **+** | **+** |
| 45_2 | 5'-nucleotidase protein [Trichuris suis] | KHJ47404.1 |  |  | **+** |  |
| 45_3 | hypothetical protein D918_04345 [Trichuris suis] | KHJ45608.1 |  |  | **c** |  |
| 45_4 | hypothetical protein M513_04837 [Trichuris suis] | KFD54295.1 |  |  | **c** |  |
| 46_1 | hypothetical protein D918_06825 [Trichuris suis] | KHJ42968.1 |  | **+** |  |  |
| 46_2 | hypothetical protein M513_08123 [Trichuris suis] | KFD50941.1 |  | **+** |  |  |
| 47_1 | hypothetical protein D918_09642 [Trichuris suis] | KHJ40336.1 |  | **+** |  |  |
| 47_2 | hypothetical protein M513_10945 [Trichuris suis] | KFD48159.1 |  | **+** |  |  |
| 47_3 | hypothetical protein M514_10945 [Trichuris suis] | KFD61896.1 |  | **+** |  |  |
| 48_1 | hypothetical protein M513_04629 [Trichuris suis] | KFD54482.1 |  | **+** |  |  |
| 48_2 | hypothetical protein M514_04629 [Trichuris suis] | KFD73347.1 |  | **+** |  |  |
| 49_1 | deoxyribonuclease II [Trichuris suis] | KHJ48080.1 |  | **+** | **c** | **c** |
| 49_2 | hypothetical protein M513_01156 [Trichuris suis] | KFD57923.1 |  | **+** |  |  |
| 49_3 | hypothetical protein M514_17021 [Trichuris suis] | KFD70899.1 |  | **+** |  |  |
| 50_1 | hypothetical protein D918_02817 [Trichuris suis] | KHJ47243.1 |  | **+** |  |  |
| 50_2 | hypothetical protein M513_13694 [Trichuris suis] | KFD45428.1 |  | **+** |  |  |
| 50_3 | hypothetical protein M514_13694 [Trichuris suis] | KFD60175.1 |  | **+** |  |  |
| 50_4 | hypothetical protein M514_25363 [Trichuris suis] | KFD62430.1 |  | **+** |  |  |
| 51_1 | hypothetical protein M513_05301 [Trichuris suis] | KFD53795.1 |  | **+** |  |  |
| 51_2 | hypothetical protein M514_05301 [Trichuris suis] | KFD71589.1 |  | **+** |  |  |
| 52_1 | eukaryotic aspartyl protease [Trichuris suis] | KHJ46445.1 |  | **+** |  |  |
| 52_2 | hypothetical protein M513_01139 [Trichuris suis] | KFD57906.1 |  | **+** |  |  |
| 52_3 | hypothetical protein M514_01139, partial [Trichuris suis] | KFD70919.1 |  | **+** |  |  |
| 53 | hypothetical protein M514_18642 [Trichuris suis] | KFD69065.1 |  | **+** | **+** | **+** |
| 54 | hypothetical protein M513_04702 [Trichuris suis] | KFD54359.1 |  | **+** | **c** | **+** |
| 55 | hypothetical protein M513_02643 [Trichuris suis] | KFD56539.1 |  | **+** | **c** | **+** |
| 56 | hypothetical protein M513_05339 [Trichuris suis] | KFD53833.1 |  | **+** | **+** | **+** |
| 57 | hypothetical protein M513_12223, partial [Trichuris suis] | KFD46901.1 |  | **+** | **+** | **+** |
| 58 | hypothetical protein D918_01923 [Trichuris suis] | KHJ47765.1 |  | **+** |  | **+** |
| 59 | hypothetical protein M513_05264, partial [Trichuris suis] | KFD53758.1 |  | **+** | **c** | **c** |
| 60 | hypothetical protein M514_27940 [Trichuris suis] | KFD59881.1 |  | **+** |  | **c** |
| 61 | hypothetical protein M513_01283 [Trichuris suis] | KFD58050.1 |  | **+** |  | **c** |
| 62 | hypothetical protein M513_08967, partial [Trichuris suis] | KFD50128.1 |  | **+** |  | **c** |
| 63 | hypothetical protein M513_01523 [Trichuris suis] | KFD57420.1 |  | **+** |  |  |
| 64 | hypothetical protein M514_10707 [Trichuris suis] | KFD62439.1 |  | **+** |  |  |
| 65 | hypothetical protein M513_06133 [Trichuris suis] | KFD53017.1 |  | **+** |  |  |
| 66 | hypothetical protein M513_03688 [Trichuris suis] | KFD55348.1 |  | **+** |  |  |
| 67 | hypothetical protein M513_01548 [Trichuris suis] | KFD57445.1 |  | **+** |  |  |
| 68 | hypothetical protein M513_10380 [Trichuris suis] | KFD48750.1 |  | **+** |  |  |
| 69 | hypothetical protein M514_15631 [Trichuris suis] | KFD72358.1 |  | **+** |  |  |
| 70 | hypothetical protein M513_05720 [Trichuris suis] | KFD53456.1 |  | **+** |  |  |
| 71 | hypothetical protein M513_13062 [Trichuris suis] | KFD46058.1 |  | **+** |  |  |
| 72 | hypothetical protein M513_09650 [Trichuris suis] | KFD49465.1 |  | **+** |  |  |
| 73 | hypothetical protein M514_11842 [Trichuris suis] | KFD60275.1 |  | **+** |  |  |
| 74 | hypothetical protein M513_09708 [Trichuris suis] | KFD49441.1 |  | **+** |  |  |
| 75 | hypothetical protein M513_13311 [Trichuris suis] | KFD45809.1 |  | **+** |  |  |
| 76_1 | hypothetical protein M514_06559 [Trichuris suis] | KFD65215.1 |  | **c** | **+** | **+** |
| 76_2 | hypothetical protein M513_06559 [Trichuris suis] | KFD52525.1 |  | **c** |  | **+** |
| 76_3 | hypothetical protein D918_09267 [Trichuris suis] | KHJ40675.1 |  | **c** |  |  |
| 77_1 | hypothetical protein D918_02980 [Trichuris suis] | KHJ46659.1 |  | **c** | **c** | **+** |
| 77_2 | hypothetical protein M513_09161 [Trichuris suis] | KFD49947.1 |  | **c** | **c** | **+** |
| 78_1 | hypothetical protein M513_07011 [Trichuris suis] | KFD52166.1 |  | **c** | **+** | **+** |
| 78_2 | hypothetical protein M514_07011 [Trichuris suis] | KFD65195.1 |  | **c** | **+** | **+** |
| 79_1 | hypothetical protein M513_11843 [Trichuris suis] | KFD47290.1 |  | **c** |  | **c** |
| 79_2 | hypothetical protein M514_11843 [Trichuris suis] | KFD60276.1 |  | **c** |  | **c** |
| 80_1 | hypothetical protein M513_08977 [Trichuris suis] | KFD50138.1 |  | **c** | **c** | **c** |
| 80_2 | hypothetical protein M514_08977 [Trichuris suis] | KFD70020.1 |  | **c** | **c** | **c** |
| 81_1 | hypothetical protein D918_01088 [Trichuris suis] | KHJ48783.1 |  | **c** |  | **c** |
| 81_2 | hypothetical protein M513_04058 [Trichuris suis] | KFD55140.1 |  | **c** |  | **c** |
| 82_1 | hypothetical protein M513_03843 [Trichuris suis] | KFD55202.1 |  | **c** | **+** | **c** |
| 82_2 | hypothetical protein M514_22380 [Trichuris suis] | KFD65454.1 |  | **c** | **+** | **c** |
| 83_1 | hypothetical protein M513_08058 [Trichuris suis] | KFD51017.1 |  | **c** | **c** | **c** |
| 83_2 | hypothetical protein M514_08058 [Trichuris suis] | KFD73230.1 |  | **c** | **c** | **c** |
| 84_1 | hypothetical protein D918_06364 [Trichuris suis] | KHJ43455.1 |  | **c** |  | **c** |
| 84_2 | hypothetical protein M513_13969 [Trichuris suis] | KFD45154.1 |  | **c** |  | **c** |
| 84_3 | hypothetical protein M514_13969 [Trichuris suis] | KFD60414.1 |  | **c** |  | **c** |
| 85 | nematode cuticle collagen domain protein [Trichuris suis] | KHJ49255.1 |  | **c** | **c** | **c** |
| 86_1 | hypothetical protein M513_13381 [Trichuris suis] | KFD45741.1 |  | **c** |  | **c** |
| 86_2 | hypothetical protein M514_13381 [Trichuris suis] | KFD69755.1 |  | **c** |  | **c** |
| 87_1 | hypothetical protein M513_13112 [Trichuris suis] | KFD46010.1 |  | **c** | **+** | **+** |
| 87_2 | hypothetical protein M514_13112 [Trichuris suis] | KFD61255.1 |  | **c** | **+** | **+** |
| 88_1 | hypothetical protein M514_01527 [Trichuris suis] | KFD66530.1 |  | **c** | **c** | **+** |
| 88_2 | hypothetical protein M513_01527 [Trichuris suis] | KFD57424.1 |  | **c** | **c** | **+** |
| 88_3 | hypothetical protein D918_00204, partial [Trichuris suis] | KHJ49086.1 |  | **c** |  |  |
| 89_1 | hypothetical protein D918_08127 [Trichuris suis] | KHJ41756.1 |  | **c** | **c** |  |
| 89_2 | hypothetical protein M513_10856 [Trichuris suis] | KFD48279.1 |  | **c** | **c** |  |
| 90_1 | hypothetical protein D918_03106 [Trichuris suis] | KHJ46759.1 |  | **c** |  |  |
| 90_2 | hypothetical protein M513_07603 [Trichuris suis] | KFD51553.1 |  | **c** |  |  |
| 90_3 | hypothetical protein M514_07603 [Trichuris suis] | KFD65633.1 |  | **c** |  |  |
| 91_1 | hypothetical protein D918_04548 [Trichuris suis] | KHJ45244.1 |  | **c** |  |  |
| 91_2 | hypothetical protein M513_03842 [Trichuris suis] | KFD55201.1 |  | **c** |  |  |
| 91_3 | hypothetical protein M514_03842 [Trichuris suis] | KFD65453.1 |  | **c** |  |  |
| 92_1 | hypothetical protein M513_03423 [Trichuris suis] | KFD55675.1 |  | **c** |  |  |
| 92_2 | WAP-type 'four-disulfide core [Trichuris suis] | KHJ48262.1 |  | **c** |  |  |
| 93_1 | cathepsin propeptide inhibitor domain protein [Trichuris suis] | KHJ41450.1 |  | **c** |  |  |
| 93_2 | hypothetical protein M513_07451 [Trichuris suis] | KFD51754.1 |  | **c** |  |  |
| 94_1 | hypothetical protein M513_11177 [Trichuris suis] | KFD47944.1 |  | **c** |  |  |
| 94_2 | hypothetical protein M514_11177, partial [Trichuris suis] | KFD64176.1 |  | **c** |  |  |
| 95_1 | hypothetical protein D918_04979 [Trichuris suis] | KHJ44744.1 |  | **c** |  |  |
| 95_2 | hypothetical protein M513_06970 [Trichuris suis] | KFD52125.1 |  | **c** |  |  |
| 95_3 | hypothetical protein M514_06970 [Trichuris suis] | KFD61632.1 |  | **c** |  |  |
| 96_1 | hypothetical protein D918_02126 [Trichuris suis] | KHJ47266.1 |  | **c** |  |  |
| 96_2 | hypothetical protein M513_02266, partial [Trichuris suis] | KFD57009.1 |  | **c** |  |  |
| 96_3 | hypothetical protein M514_02266, partial [Trichuris suis] | KFD70106.1 |  | **c** |  |  |
| 97_1 | hypothetical protein D918_04420 [Trichuris suis] | KHJ45116.1 |  | **c** |  |  |
| 97_2 | hypothetical protein M513_09697 [Trichuris suis] | KFD49430.1 |  | **c** |  |  |
| 97_3 | hypothetical protein M514_24178 [Trichuris suis] | KFD63646.1 |  | **c** |  |  |
| 98 | hypothetical protein M513_05014 [Trichuris suis] | KFD53995.1 |  | **c** | **+** | **+** |
| 99 | hypothetical protein M513_07304 [Trichuris suis] | KFD51777.1 |  | **c** |  | **+** |
| 100 | hypothetical protein M513_01916 [Trichuris suis] | KFD57031.1 |  | **c** | **c** | **c** |
| 101 | hypothetical protein M513_10504 [Trichuris suis] | KFD48649.1 |  | **c** |  | **+** |
| 102 | hypothetical protein M513_13254 [Trichuris suis] | KFD45864.1 |  | **c** |  | **+** |
| 103 | hypothetical protein M513_02878 [Trichuris suis] | KFD56100.1 |  | **c** | **c** | **c** |
| 104 | hypothetical protein M513_07012 [Trichuris suis] | KFD52167.1 |  | **c** | **c** |  |
| 105 | Carboxylesterase [Trichuris suis] | KHJ48427.1 |  | **c** |  |  |
| 106 | hypothetical protein M513_13834 [Trichuris suis] | KFD45288.1 |  | **c** |  |  |
| 107 | hypothetical protein M513_00705 [Trichuris suis] | KFD58479.1 |  | **c** |  |  |
| 108 | hypothetical protein M513_00638 [Trichuris suis] | KFD58412.1 |  | **c** |  |  |
| 109 | hypothetical protein M513_01343, partial [Trichuris suis] | KFD57673.1 |  | **c** |  |  |
| 110 | hypothetical protein M513_02830 [Trichuris suis] | KFD56375.1 |  | **c** |  |  |
| 111 | hypothetical protein M513_04328 [Trichuris suis] | KFD54894.1 |  | **c** |  |  |
| 112_1 | hypothetical protein M513_07066 [Trichuris suis] | KFD52084.1 |  |  | **+** | **+** |
| 112_2 | hypothetical protein M514_07066 [Trichuris suis] | KFD65846.1 |  |  | **+** | **+** |
| 113_1 | hypothetical protein M513_03361 [Trichuris suis] | KFD55613.1 |  |  | **+** | **+** |
| 113_2 | hypothetical protein M514_03361 [Trichuris suis] | KFD68024.1 |  |  | **+** | **+** |
| 114_1 | hypothetical protein M513_08330 [Trichuris suis] | KFD50789.1 |  |  | **c** | **+** |
| 114_2 | hypothetical protein M514_08330 [Trichuris suis] | KFD63404.1 |  |  | **c** | **+** |
| 115_1 | hypothetical protein M513_13670 [Trichuris suis] | KFD45454.1 |  |  | **c** | **+** |
| 115_2 | hypothetical protein M514_13670 [Trichuris suis] | KFD67510.1 |  |  | **c** | **+** |
| 116_1 | hypothetical protein M513_01531 [Trichuris suis] | KFD57428.1 |  |  | **+** | **+** |
| 116_2 | hypothetical protein M514_01531 [Trichuris suis] | KFD66534.1 |  |  | **+** | **+** |
| 117_1 | hypothetical protein D918_07287 [Trichuris suis] | KHJ42568.1 |  |  | **c** | **+** |
| 117_2 | hypothetical protein M513_03657 [Trichuris suis] | KFD55317.1 |  |  | **c** | **+** |
| 117_3 | hypothetical protein M514_03657 [Trichuris suis] | KFD59705.1 |  |  | **c** | **+** |
| 118_1 | hypothetical protein M513_10502 [Trichuris suis] | KFD48647.1 |  |  | **+** | **+** |
| 118_2 | hypothetical protein M514_10502 [Trichuris suis] | KFD62239.1 |  |  | **+** | **+** |
| 119_1 | hypothetical protein D918_06254 [Trichuris suis] | KHJ43749.1 |  |  |  | **+** |
| 119_2 | hypothetical protein M513_06011 [Trichuris suis] | KFD53097.1 |  |  |  | **+** |
| 119_3 | hypothetical protein M514_06012 [Trichuris suis] | KFD70755.1 |  |  |  | **c** |
| 120_1 | hypothetical protein M513_07662 [Trichuris suis] | KFD51449.1 |  |  | **c** | **+** |
| 120_2 | hypothetical protein M514_07662 [Trichuris suis] | KFD65775.1 |  |  | **c** | **+** |
| 121_1 | hypothetical protein D918_00205 [Trichuris suis] | KHJ49087.1 |  |  | **c** | **c** |
| 121_2 | hypothetical protein M513_01529 [Trichuris suis] | KFD57426.1 |  |  | **c** | **+** |
| 121_3 | hypothetical protein M514_01529 [Trichuris suis] | KFD66532.1 |  |  | **c** | **+** |
| 122_1 | hypothetical protein M513_01155 [Trichuris suis] | KFD57922.1 |  |  | **c** | **c** |
| 122_2 | hypothetical protein M514_17022 [Trichuris suis] | KFD70900.1 |  |  | **c** | **c** |
| 123_1 | hypothetical protein M513_05968 [Trichuris suis] | KFD53054.1 |  |  |  | **c** |
| 123_2 | hypothetical protein M513_05970 [Trichuris suis] | KFD53056.1 |  |  |  | **c** |
| 123_3 | hypothetical protein M514_27538 [Trichuris suis] | KFD60285.1 |  |  |  | **c** |
| 124_1 | hypothetical protein M513_09721 [Trichuris suis] | KFD49358.1 |  |  |  | **c** |
| 124_2 | hypothetical protein M514_13659 [Trichuris suis] | KFD64576.1 |  |  |  | **c** |
| 125_1 | hypothetical protein D918_04914 [Trichuris suis] | KHJ44680.1 |  |  |  | **c** |
| 125_2 | hypothetical protein M513_12191 [Trichuris suis] | KFD46945.1 |  |  |  | **c** |
| 125_3 | hypothetical protein M514_12191 [Trichuris suis] | KFD62216.1 |  |  |  | **c** |
| 126_1 | hypothetical protein D918_05935 [Trichuris suis] | KHJ43882.1 |  |  |  | **c** |
| 126_2 | hypothetical protein M513_02959 [Trichuris suis] | KFD56181.1 |  |  |  | **c** |
| 127_1 | hypothetical protein D918_02591 [Trichuris suis] | KHJ47045.1 |  |  | **+** | **c** |
| 127_2 | hypothetical protein M514_05551, partial [Trichuris suis] | KFD62628.1 |  |  | **+** | **c** |
| 128_1 | collagen triple helix repeat protein [Trichuris suis] | KHJ49597.1 |  |  | **c** | **c** |
| 128_2 | hypothetical protein M513_08053 [Trichuris suis] | KFD51012.1 |  |  | **c** | **c** |
| 129_1 | hypothetical protein M513_06427 [Trichuris suis] | KFD52771.1 |  |  |  | **c** |
| 129_2 | hypothetical protein M514_27457, partial [Trichuris suis] | KFD60372.1 |  |  |  | **c** |
| 130_1 | hypothetical protein D918_08193 [Trichuris suis] | KHJ41663.1 |  |  | **c** | **c** |
| 130_2 | hypothetical protein M513_06801 [Trichuris suis] | KFD52238.1 |  |  | **c** | **c** |
| 130_3 | hypothetical protein M514_06801 [Trichuris suis] | KFD66698.1 |  |  | **c** | **c** |
| 131_1 | hypothetical protein D918_00794 [Trichuris suis] | KHJ48492.1 |  |  |  | **c** |
| 131_2 | hypothetical protein M513_09606 [Trichuris suis] | KFD49495.1 |  |  |  | **c** |
| 131_3 | hypothetical protein M514_09606 [Trichuris suis] | KFD65669.1 |  |  |  | **c** |
| 132_1 | hypothetical protein M513_02956 [Trichuris suis] | KFD56178.1 |  |  | **+** | **+** |
| 132_2 | Kunitz/Bovine pancreatic trypsin inhibitor domain protein [Trichuris suis] | KHJ47348.1 |  |  | **+** | **+** |
| 133_1 | hypothetical protein D918_03205 [Trichuris suis] | KHJ46832.1 |  |  | **+** | **+** |
| 133_2 | hypothetical protein M513_13363 [Trichuris suis] | KFD45757.1 |  |  | **c** | **+** |
| 133_3 | hypothetical protein M514_20584 [Trichuris suis] | KFD67148.1 |  |  | **c** | **+** |
| 134_1 | hypothetical protein M513_07111 [Trichuris suis] | KFD51979.1 |  |  |  | **+** |
| 134_2 | hypothetical protein M513_14229, partial [Trichuris suis] | KFD44894.1 |  |  |  | **+** |
| 134_3 | hypothetical protein M514_07111 [Trichuris suis] | KFD71407.1 |  |  |  | **+** |
| 135_1 | hypothetical protein M513_11184 [Trichuris suis] | KFD47951.1 |  |  |  | **c** |
| 135_2 | hypothetical protein M514_28192 [Trichuris suis] | KFD59628.1 |  |  |  | **c** |
| 136_1 | hypothetical protein D918_03945 [Trichuris suis] | KHJ45733.1 |  |  |  | **c** |
| 136_2 | hypothetical protein M513_01096 [Trichuris suis] | KFD57863.1 |  |  |  | **c** |
| 137_1 | hypothetical protein M513_13760 [Trichuris suis] | KFD45364.1 |  |  |  | **c** |
| 137_2 | hypothetical protein M514_27479 [Trichuris suis] | KFD60349.1 |  |  |  | **c** |
| 137_3 | molybdenum cofactor biosynthesis protein A [Trichuris suis] | KHJ46840.1 |  |  |  | **c** |
| 138_1 | hypothetical protein M514_12087 [Trichuris suis] | KFD67515.1 |  |  | **+** | **+** |
| 138_2 | hypothetical protein M514_24411 [Trichuris suis] | KFD63390.1 |  |  | **+** | **+** |
| 138_3 | hypothetical protein M513_12087 [Trichuris suis] | KFD47044.1 |  |  | **+** |  |
| 139_1 | hypothetical protein M513_13529 [Trichuris suis] | KFD45594.1 |  |  | **c** | **+** |
| 139_2 | hypothetical protein D918_01462 [Trichuris suis] | KHJ48194.1 |  |  | **c** |  |
| 140_1 | hypothetical protein M513_10412 [Trichuris suis] | KFD48701.1 |  |  | **c** |  |
| 140_2 | hypothetical protein M514_10412, partial [Trichuris suis] | KFD69291.1 |  |  | **c** |  |
| 141_1 | hypothetical protein D918_00022 [Trichuris suis] | KHJ48917.1 |  |  | **c** |  |
| 141_2 | hypothetical protein M513_07110, partial [Trichuris suis] | KFD51978.1 |  |  | **c** |  |
| 141_3 | hypothetical protein M514_07110, partial [Trichuris suis] | KFD71408.1 |  |  | **c** |  |
| 142_1 | hypothetical protein M514_18178 [Trichuris suis] | KFD69562.1 |  |  | **+** | **+** |
| 142_2 | hypothetical protein M513_01929 [Trichuris suis] | KFD57044.1 |  |  | **c** |  |
| 143 | hypothetical protein M513_11423 [Trichuris suis] | KFD47690.1 |  |  | **+** | **+** |
| 144 | hypothetical protein M513_13284 [Trichuris suis] | KFD45838.1 |  |  | **+** | **+** |
| 145 | hypothetical protein M514_00541 [Trichuris suis] | KFD67474.1 |  |  |  | **+** |
| 146 | hypothetical protein M514_26563 [Trichuris suis] | KFD61265.1 |  |  |  | **+** |
| 147 | hypothetical protein M513_00573 [Trichuris suis] | KFD58347.1 |  |  | **c** | **+** |
| 148 | hypothetical protein M513_12471 [Trichuris suis] | KFD46662.1 |  |  | **c** | **+** |
| 149 | hypothetical protein D918_02746 [Trichuris suis] | KHJ47180.1 |  |  | **+** | **+** |
| 150 | hypothetical protein D918_01432 [Trichuris suis] | KHJ48165.1 |  |  | **+** | **+** |
| 151 | hypothetical protein M513_13496 [Trichuris suis] | KFD45624.1 |  |  | **+** | **+** |
| 152 | hypothetical protein M513_11596 [Trichuris suis] | KFD47502.1 |  |  | **+** | **+** |
| 153 | hypothetical protein M514_27849, partial [Trichuris suis] | KFD59977.1 |  |  | **+** | **+** |
| 154 | hypothetical protein M513_12184 [Trichuris suis] | KFD46938.1 |  |  |  | **+** |
| 155 | hypothetical protein M513_03550 [Trichuris suis] | KFD55498.1 |  |  | **+** | **+** |
| 156 | hypothetical protein M513_14359, partial [Trichuris suis] | KFD44764.1 |  |  |  | **+** |
| 157 | hypothetical protein M514_27845 [Trichuris suis] | KFD59973.1 |  |  | **c** | **+** |
| 158 | hypothetical protein M513_05101 [Trichuris suis] | KFD54082.1 |  |  |  | **+** |
| 159 | hypothetical protein M513_06715 [Trichuris suis] | KFD52334.1 |  |  | **+** | **+** |
| 160 | hypothetical protein M513_06707 [Trichuris suis] | KFD52326.1 |  |  | **c** | **+** |
| 161 | hypothetical protein M514_17400 [Trichuris suis] | KFD70316.1 |  |  |  | **c** |
| 162 | hypothetical protein M513_07352 [Trichuris suis] | KFD51825.1 |  |  |  | **c** |
| 163 | hypothetical protein M514_11217 [Trichuris suis] | KFD67997.1 |  |  |  | **c** |
| 164 | hypothetical protein M513_05021 [Trichuris suis] | KFD54002.1 |  |  |  | **c** |
| 165 | hypothetical protein M513_01675 [Trichuris suis] | KFD57572.1 |  |  | **c** | **c** |
| 166 | hypothetical protein M513_10507 [Trichuris suis] | KFD48652.1 |  |  |  | **c** |
| 167 | hypothetical protein M514_27025 [Trichuris suis] | KFD60805.1 |  |  |  | **c** |
| 168 | hypothetical protein M514_24426, partial [Trichuris suis] | KFD63405.1 |  |  |  | **+** |
| 169 | hypothetical protein M513_13457 [Trichuris suis] | KFD45663.1 |  |  |  | **c** |
| 170 | hypothetical protein M513_07989 [Trichuris suis] | KFD51089.1 |  |  |  | **c** |
| 171 | hypothetical protein M513_00299, partial [Trichuris suis] | KFD58606.1 |  |  |  | **c** |
| 172 | hypothetical protein M513_03825 [Trichuris suis] | KFD55184.1 |  |  |  | **c** |
| 173 | hypothetical protein M513_01736 [Trichuris suis] | KFD57225.1 |  |  |  | **c** |
| 174 | hypothetical protein M513_10508 [Trichuris suis] | KFD48653.1 |  |  | **c** | **c** |
| 175 | hypothetical protein M513_05511 [Trichuris suis] | KFD53595.1 |  |  | **c** |  |
|  |  |  |  |  |  |  |
|  |  |  |  |  |  |  |
